# Integrated transcriptomic and metabolomic analysis reveals the metabolic programming of GM-CSF- and M-CSF- differentiated mouse macrophages

**DOI:** 10.3389/fimmu.2023.1230772

**Published:** 2023-09-25

**Authors:** Qianyue Zhang, Qiaoling Song, Shan Liu, Yuting Xu, Danling Gao, Peizhe Lu, Yuantao Liu, Guanghui Zhao, Lihong Wu, Chenyang Zhao, Jinbo Yang

**Affiliations:** ^1^ Key Laboratory of Marine Drugs, Ministry of Education of China, School of Medicine and Pharmacy, Ocean University of China, Qingdao, China; ^2^ Innovation Platform of Marine Drug Screening and Evaluation, Qingdao Marine Science and Technology Center, Qingdao, China; ^3^ Department of Neuroscience, University of Michigan, Ann Arbor, MI, United States; ^4^ Department of Endocrinology, Qilu Hospital (Qingdao), Cheeloo College of Medicine, Shandong University, Qingdao, China; ^5^ Medical Laboratory Center, Qilu Hospital (Qingdao), Cheeloo College of Medicine, Shandong University, Qingdao, China; ^6^ Oncology Laboratory, Qilu Hospital (Qingdao), Cheeloo College of Medicine, Shandong University, Qingdao, China

**Keywords:** macrophage differentiation, GM-CSF, M-CSF, immunometabolism, transcriptome, metabolome

## Abstract

Macrophages play a critical role in the inflammatory response and tumor development. Macrophages are primarily divided into pro-inflammatory M1-like and anti-inflammatory M2-like macrophages based on their activation status and functions. *In vitro* macrophage models could be derived from mouse bone marrow cells stimulated with two types of differentiation factors: GM-CSF (GM-BMDMs) and M-CSF (M-BMDMs), to represent M1- and M2-like macrophages, respectively. Since macrophage differentiation requires coordinated metabolic reprogramming and transcriptional rewiring in order to fulfill their distinct roles, we combined both transcriptome and metabolome analysis, coupled with experimental validation, to gain insight into the metabolic status of GM- and M-BMDMs. The data revealed higher levels of the tricarboxylic acid cycle (TCA cycle), oxidative phosphorylation (OXPHOS), fatty acid oxidation (FAO), and urea and ornithine production from arginine in GM-BMDMs, and a preference for glycolysis, fatty acid storage, bile acid metabolism, and citrulline and nitric oxide (NO) production from arginine in M-BMDMs. Correlation analysis with the proteomic data showed high consistency in the mRNA and protein levels of metabolic genes. Similar results were also obtained when compared to RNA-seq data of human monocyte derived macrophages from the GEO database. Furthermore, canonical macrophage functions such as inflammatory response and phagocytosis were tightly associated with the representative metabolic pathways. In the current study, we identified the core metabolites, metabolic genes, and functional terms of the two distinct mouse macrophage populations. We also distinguished the metabolic influences of the differentiation factors GM-CSF and M-CSF, and wish to provide valuable information for *in vitro* macrophage studies.

## 1 Introduction

Macrophages are highly plastic cells with distinct phenotypes. They respond rapidly to environmental signals and are essential for host defense, tissue homeostasis and repair, pathology, and development (1). Over the past few decades, several *in vitro* models have been applied to study macrophage differentiation and polarization. Bone marrow-derived macrophages (BMDMs) primed with the differentiation factors GM-CSF (GM-BMDMs, GM) and M-CSF (M-BMDMs, M) are one of the most commonly used macrophage models (2). M-CSF is known for its role in inducing macrophage differentiation towards an anti-inflammatory phenotype (M2-like macrophages), whereas GM-CSF induces macrophage differentiation towards a pro-inflammatory phenotype (M1-like macrophages) (3, 4). In recent years, a number of studies have been reported in an effort to fully elucidate the various aspects of BMDMs (5–7). For example, the key chromatin modulators (e.g. H3K27me3) (5) and essential signaling pathways (e.g. type I interferon signaling) (8, 9) for macrophage differentiation, the influence of culture conditions on inflammatory responses (6), and the heterogeneity of macrophage populations have been thoroughly described in literature (10). To better understand macrophage plasticity, more attention has been placed on orchestrating the course of macrophage differentiation.

In recent years, there has been an increasing focus on metabolism in the study of immune cells due to its key involvement in the regulation of cell fate and function (immunometabolism) (11–13). Metabolic pathways have great potential to regulate or support functional changes, as transitions between quiescent and activated states require the involvement of metabolites in different pathways (11). Due to the high plasticity of macrophages, it is essential to elucidate the immunometabolic status of different types of macrophages both *in vitro* and *in vivo*. To date, numerous in-depth analyses have been performed to illustrate the metabolic status of activated macrophages with different polarization status: M-CSF-primed macrophages polarized by LPS/IFNγ for M1 and by IL4/IL13 for M2 (12, 13). Collectively, LPS/IFNγ polarized M1 macrophages exhibit increased glycolysis, fatty acid synthesis (FAS), arginine conversion to nitric oxide (NO) by iNOS (13, 14), whereas IL4/IL13 polarized M2a macrophages are more dependent on oxidative phosphorylation (OXPHOS), fatty acid oxidation (FAO), and arginine conversion to urea and ornithine to maintain their polarization status *in vitro* (13, 14). Tumor associated macrophages (TAMs, M2d type) exhibit enhanced aerobic glycolysis, FAS, and intracellular glutamine levels *in vivo* (15).

Although the metabolic changes of macrophages with different polarization status have been extensively studied, there are few studies elucidating the immunometabolic characteristics of GM-CSF and M-CSF differentiated/matured macrophages (16–18). By performing proteomic analysis of mouse bone marrow cells cultured with GM-CSF or L929 conditioned medium (CM) (source of M-CSF), the glycolytic capacity, as well as the nitrogen compound biosynthesis, was enhanced in GM-CSF primed BMDMs (16). However, L929-CM primed BMDMs have increased mitochondrial mass, and exhibit higher glycolysis and oxygen consumption response to LPS challenge when compared with M-CSF primed BMDMs (19). Using murine peritoneal macrophages (M0) treated with GM-CSF and M-CSF for a relatively short time (2 days instead of 7 days for BMDMs), induced gene expression for glucose metabolism and mitochondrial biogenesis was demonstrated in both GM and M macrophages, with a preferential overexpression of several critical glycolytic enzymes in M compared to GM macrophages (17). In human monocyte-derived macrophages (MDMs), but not BMDMs primed with GM-CSF and M-CSF, a higher oxygen consumption and aerobic glycolysis in GM, and upregulation of glycolytic enzyme gene expression were also observed (18). Due to the differences among these employed models including culture conditions, source of growth factors, and cell species, there is still no clear conclusive description of the representative metabolic status of GM-CSF and M-CSF differentiating macrophages, especially for the metabolic pathways other than glycolytic process. Therefore, it’s urgent and valuable to elucidate the global landscape of metabolic phenotype of mature GM and M macrophages without additional stimuli such as cytokines, metabolites, and microbial ligands.

In this study, we performed both metabolic and transcriptomic analysis on mouse M-CSF and GM-CSF primed BMDMs. Numerous metabolites are significantly regulated, and, together with differentially expressed genes, jointly describe the metabolic characteristics of GM and M. The tricarboxylic acid cycle (TCA cycle) and OXPHOS are preferred by GM macrophages for glucose metabolism, while FAO is also preferred for additional acetyl coenzyme A (CoA) generation for the TCA cycle due to their robust mitochondrial function. Nevertheless, M macrophages prefer glycolysis, fatty acid storage, and bile acid metabolism. The uptake of essential amino acids such as leucine, methionine and phenylalanine is often active in GM. Arginine is metabolized into urea and ornithine by arginase (Arg) in GM, whereas citrulline and NO are the arginine metabolites in M. Collectively, our results may highlight the metabolic landscape of GM-CSF- and M-CSF- differentiated mouse macrophages and attempt to establish the framework for further metabolism-based studies of macrophage plasticity.

## 2 Methods

### 2.1 Reagents

TIANamp Genomic DNA Kit (Cat. DP304) was from Tiangen. SYBR Green PCR Master Mix (2×) (Cat.4913914001) was from Roche. Recombinant mouse M-CSF (Cat. 415-ML) and GM-CSF (Cat. 416-ML) were purchased from R&D Systems. Glycolytic Rate Assay Kit (Cat. 103346-100), XF cell Mito Stress Test Kit (Cat. 103010-100) and Cell Energy Phenotype Test Kit (Cat. 103275-100) were from Agilent. Glucose Assay Kit (Cat. AB169559), Citrulline Fluorometric Assay Kit (Cat. AB273309), Ornithine Fluorometric Assay Kit (Cat. AB252903), and Adipogenesis Colorimetric/Fluorometric Assay Kit (Cat. AB102513) were form Abcam. Nitric Oxide Assay Kit (Cat. S0021S), Nitric Oxide Synthase Assay Kit (Cat. S0025) and Mito-Tracker Green (Cat. C1048) was from Beyotime. Bis Benzimide Hoechst NO33342 (Cat. B8040) was from Solarbio. Arginase Activity Assay Kit (Cat. MAK112) was from Sigma-Aldrich.

### 2.2 Animals

C57BL/6J mice (SPF grade, male, 6-10 weeks old) were purchased from Beijing Vital River Laboratory Animal Technology and maintained in a temperature- and humidity- controlled room with a 12-hour light-dark cycle. All animal procedures were approved by the Committee of Experimental Animals of School of Medicine and Pharmacy, Ocean University of China (OUC-SMP-2019-02-02).

### 2.3 Bone marrow derived macrophage culture

Mouse BMDMs were prepared as previously described (6). Briefly, bone marrow cells were collected from femurs. Cell suspensions were passed through a 100 μm cell strainer, collected by centrifugation at 300 g for 10 minutes, and resuspended in DMEM containing 10% FBS, 100 U/ml penicillin, 100 μg/ml streptomycin, with either 50 ng/ml M-CSF or 20 ng/ml GM-CSF. Cells were cultured at 37°C with 5% CO2 for 7 days without changing the media throughout the maturation process.

### 2.4 Transcriptome sequencing (RNA-Seq analysis)

Total RNA of mouse BMDMs was extracted using Trizol reagent kit (Invitrogen) according to the manufacturer’s protocol. RNA quality was assessed on an Agilent 2100 Bioanalyzer (Agilent) and verified by RNase free agarose gel electrophoresis. The mRNA was enriched by Oligo (dT) beads, fragmented using fragmentation buffer and reverse transcribed into cDNA using random primers. The resulting cDNA library was sequenced on an Illumina HiSeq2500 platform. Clean reads were mapped to the mouse reference genome using HISAT2.2.4 (20). The FPKM values were calculated to quantify the expression abundance and variations using StringTie software. Differential expression analysis was performed using DESeq2 software. The genes with the parameters of *P* value < 0.05 and absolute fold change (FC) > 2 were considered as differentially expressed genes (DEGs). KEGG pathway (21), GSEA (22), PPI (23), IPA (24) and gene expression correlation analysis were performed, respectively. The scaled FPKM values were normalized using the z-score approach (z = (x-m)/s), where x is the FPKM value, m is the mean of the FPKM values of a given gene in all tested samples (row mean), and s is the standard deviation of the row values. The RNA-seq data were deposited in the Gene Expression Omnibus (GEO) database under the accession number GSE198821.

### 2.5 Extraction, quantitative and qualitative analysis of metabolites

BMDMs were collected, lyophilized, and dissolved in methanol. Samples were concentrated to dry under vacuum and dissolved with 2-chlorobenzalanine (4 ppm) 80% methanol solution. Chromatographic separation was accomplished in an UHPLC (1290 Infinity LC, Agilent Technologies) equipped with an ACQUITY UPLC BEH Amide (1.7 µm, 2.1 mm x 100 mm; Waters) column maintained at 25°C. The MS experiments were executed on an AB Triple TOF 6600 mass spectrometer. The format of the raw data files was converted to mzXML format using Proteowizard (v3.0.8787). R (v3.3.2) XCMS package was used to perform peak identification, peak filtration and peak alignment for each metabolite. Mass to charge ratio (m/z), retention time and intensity, positive and negative precursor molecule were used to for subsequent analysis. Peak intensities were batch normalized to the total spectral intensity. Multivariate statistical analysis OPLS-DA was used to screen the metabolites with significant differences, and the threshold of significant differences was VIP > 1 and *P* value < 0.05. KEGG was used for the enrichment analysis of the significantly different metabolites. The metabolite data were deposited in the MetaboLights database under the accession number MTBLS6502.

### 2.6 Combined metabolomic and transcriptomic analysis

KEGG pathway maps (21) are the linking of genomic or transcriptomic contents of genes to chemical structures of endogenous molecules to perform integration analysis of genes and metabolites. All differentially expressed genes and metabolites in this study were mapped to the KEGG pathway database to obtain their links in metabolic pathways.

### 2.7 Seahorse assay

Bone marrow cells were isolated and seeded into Seahorse XF24 microplates (2.5×10^4^ GM-BMDMs and 1.5×10^4^ M-BMDMs) and incubated for 7 days as described above. The Seahorse Xfe24 Analyzer (Agilent) was used to measure proton efflux rate (PER), oxygen consumption rate (OCR), and extracellular acidification rate (ECAR) using the Glycolytic Rate Assay Kit, XF Cell Mito Stress Test Kit, and Cell Energy Phenotype Test Kit according to the manufacturer’s instructions. For the Glycolytic Rate Assay Kit, basal glycolysis was measured under basal condition before the addition of rotenone plus and antimycin A (Rot/AA) (inhibitors of complexes I and III of the mitochondrial electron transport chain). Compensatory glycolysis was determined by subtracting the rate of glycolysis before and after the addition of 2-deoxy-D-glucose (2-DG), a glucose analog that inhibits glycolysis by competitive binding of glucose hexokinase. For the XF Cell Mito Stress Test Kit, OCR was measured under basal condition and after the addition of the following drugs: 1 μM oligomycin, to inhibit mitochondrial ATP synthase; 1.5 μM fluorocarbonyl cyanide phenylhydrazone (FCCP), a protonophore that uncouples ATP synthesis from oxygen consumption by the electron-transport chain; and 100 nM rotenone plus 1 μM antimycin A (Rot/AA). For the Cell Energy Phenotype Test Kit, OCR and ECAR were measured before and after the injection of the mixture of oligomycin and FCCP.

### 2.8 Analysis of mitochondrial activity

Mito-Tracker Green (Beyotime), a lipophilic, selective dye that can be concentrated by active mitochondria, was used for mitochondrial labeling. BMDMs were incubated in 40 nM Mito-Tracker Green for 30 min at 37°C, subsequently washed with PBS. Then Hoechst33342 (Solarbio) was used for nuclear labeling. The mitochondrial morphology was reflected by green fluorescence and the nucleus by cyan fluorescence by using a high-quality cell imaging instrument (PerkinElmer). Fluorescence quantification was measured using Image J software.

### 2.9 Relative mtDNA determination

Total genomic DNA was isolated using TIANamp Genomic DNA Kit (Tiangen) following the manufacturer’s instructions. The concentration, integrity, and purity of DNAs were quantified on a Nanodrop (ND-ONE) spectrophotometer (Thermo Scientific). Real-time PCR was performed with an ABI Stepone Plus system to quantify the DNA content using SYBR Green PCR Master Mix (Roche), the mitochondrial DNA (mtDNA)-encoded genes mt-Co1 were normalized to the nuclear DNA (nDNA)-encoded gene Ndufv1, and the relative mtDNA content was determined using the 2*2^(-ΔCt) relative quantification method (25). Specific primers used in RT-PCR were as follows: mt-Nd1 forward: CTAATCGCCATAGCCTTCCTAA; mt-Nd1 reverse: GTTGTTAAAGGGCGTATTGGTT; β-actin forward: TCCTCCTGAGCGCAAGTACTCT; β-actin reverse: GCTCAGTAACAGTCCGCCTAGAA.

### 2.10 Statistical analysis

Statistical analyses were performed with Prism version 9. Data were presented graphically as the mean ± SEM. Statistical significance of differences between indicated samples was determined by unpaired Student’s t-test or two-way ANOVA. *P* value < 0.05 was considered significant and indicated by *.

## 3 Results

### 3.1 Transcriptome and metabolome jointly reveal different metabolic states between GM and M

**Figure 1:**
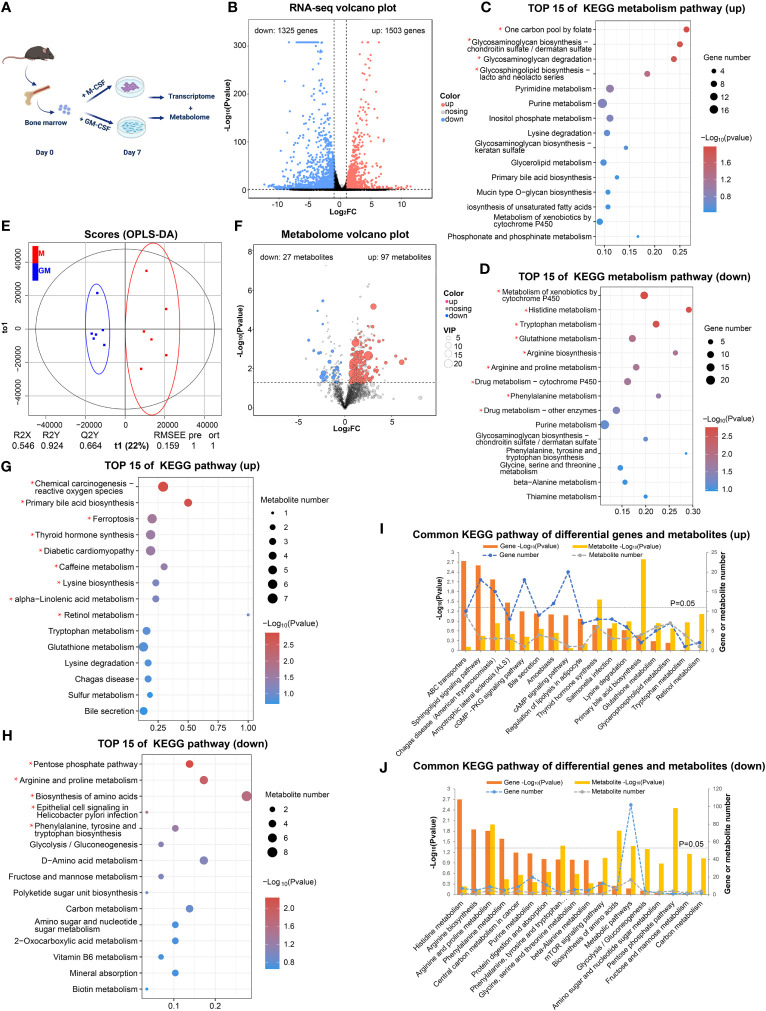
Transcriptome and metabolome jointly reveal different metabolic states between GM and M. **(A)** Research strategy for transcriptome and metabolome of GM and M. GM and M were cultured for 7 days and were processed for RNA-seq analysis (n=2) and metabolomic analysis (n=6). **(B)** The volcano plot of DEGs (FC > 2 and *P* < 0.05) in GM vs M. The black dots represent genes without significant difference between two groups; the red dots stand for significantly upregulated DEGs in M and blue dots stand for significantly downregulated ones. **(C, D)** The top 15 KEGG metabolic pathways of upregulated DEGs **(C)** and downregulated DEGs **(D)** in GM vs M. **(E)** OPLS-DA score charts combining positive and negative ion mode in metabolomic analysis. The red dots represent M samples, and the blue dots represent GM samples. **(F)** The volcano plots of differential metabolites (*P* < 0.05 and VIP > 1) in GM vs M. The grey dots represent metabolites without significant difference between two groups, the red dots represent significantly upregulated metabolites in M and the blue dots represent significantly downregulated ones. **(G, H)** The top 15 KEGG pathway analysis of upregulated differential metabolites **(G)** and downregulated differential metabolites **(H)** in GM vs M. For **(C, D)** and **(G, H)**, * indicates *P*<0.05. **(I, J)** Common pathways of KEGG enrichment analysis of upregulated **(I)** and downregulated **(J)** DEGs and differential metabolites between GM and M.

In order to comprehensively analyze the metabolic status of GM and M, we employed RNA-seq analysis to explore the expression of genes involved in metabolism and untargeted metabolome analysis to discover detailed intermediate metabolites (**Figure 1A**). According to our RNA-seq analysis (GSE198821), the gene expressions of GM and M were tightly and differentially regulated (**Figure 1B**). There were 1503 significantly upregulated and 1325 significantly downregulated differentially expressed genes (DEGs) in M compared with their GM counterparts (**Figure 1B**). KEGG analysis of these 1503 upregulated and 1325 downregulated DEGs uncovered distinct functions of M and GM (**Figures S1A, B**). To further identify the metabolic distinction between M and GM, we specifically sought out the metabolic pathways from the KEGG A class. As shown in **Figures 1C, D**, one carbon pool by folate, glycosaminoglycan biosynthesis, and glycosaminoglycan degradation, which belong to carbohydrate metabolism, was enriched in M with significance (P < 0.05) (**Figure 1C**), whereas the functions of GM were inclined toward amino acid metabolic pathways such as histidine metabolism, tryptophan metabolism, and arginine biosynthesis (**Figure 1D**).

Next, untargeted metabolome analysis was performed to distinguish the exact differences in metabolites between GM and M samples (MTBLS6502). Multivariate statistical analysis (OPLS-DA) indicated that the metabolites from M and GM were significantly different (**Figure 1E**). There were 687 significant differential metabolites in M when compared with GM, with 97 upregulated and 27 downregulated differential metabolites that could be identified in HMDB, MassBank, or METLIN databases (**Figure 1F**). KEGG pathway analysis for these differential metabolites was conducted (**Figures 1G, H**). The metabolic pathways such as primary bile acid biosynthesis, ferroptosis, lysine biosynthesis, and alpha-linolenic acid metabolism were enriched in upregulated differential metabolites, representing the metabolic functions of M (**Figure 1G**), while metabolic pathways such as pentose phosphate pathway (PPP), arginine and proline metabolism, and phenylalanine, tyrosine and tryptophan biosynthesis were enriched in downregulated differential metabolites, representing the metabolic functions of GM (**Figure 1H**). We also performed combined metabolomic and transcriptomic analysis to explore the dominant metabolic pathways in each type of BMDMs (**Figures 1I, J**). Arginine and proline metabolism was enriched in both the upregulated genes and metabolites of GM group (**Figure 1J**). For other metabolic pathways, neither gene expression nor metabolites could individually illustrate the metabolic landscape of BMDMs, and the integrated RNA-seq and metabolism analysis could be essential for further comprehensive description.

These data preliminarily suggest that prominent metabolic differences exist between GM and M with a tendency for primary bile acid biosynthesis and carbohydrate metabolism in M and active amino acid metabolism in GM.

### 3.2 High dependence on glycolysis for M and high-intensity glucose oxidation for GM

**Figure 2:**
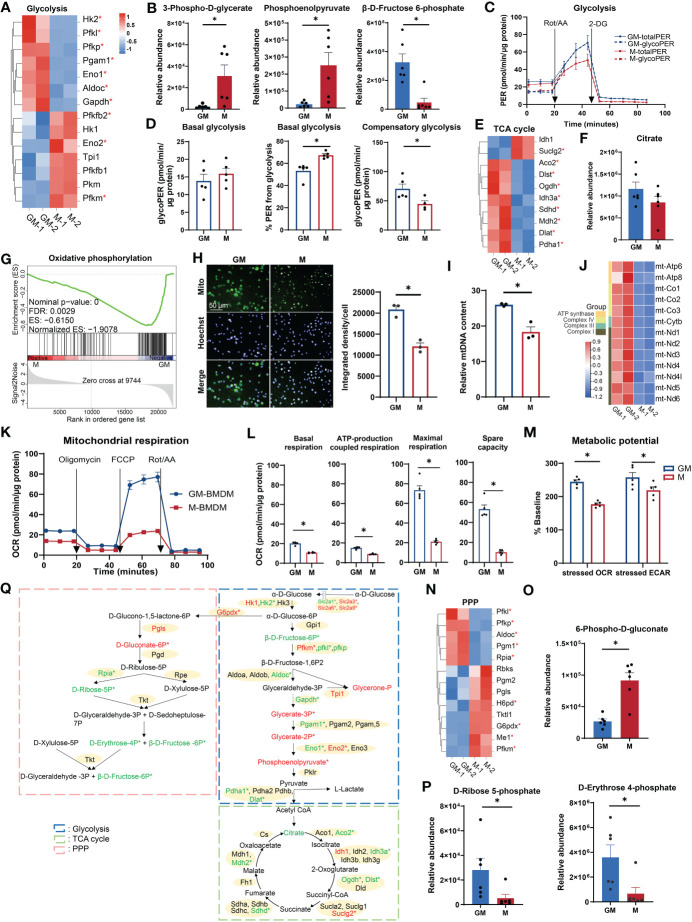
Comparison of glucose metabolism between GM and M. **(A, E, N)** Hierarchical cluster analysis for the gene expression for glycolysis (PATHER pathway database) **(A)**, TCA cycle (PATHER pathway and KEGG databases) **(E)**, and PPP (KEGG database) **(N)**. Color scale represents scaled FPKM values normalized using the z-score approach. These genes were chosen from complete genes list by cutoff |log_2_FC| > 0.25. * indicates *P* < 0.05. **(B, F, O, P)** Relative abundance of glycolysis **(B)**, TCA cycle **(F)**, PPP oxidative phase **(O)**, and non-oxidative phase **(P)** metabolites measured by LC-MS. n=6 for each group. **(C)** PER of GM and M when they were sequentially treated with Rot/AA (inhibitors of mitochondrial electron transport chain) and 2-DG (inhibitors of glycolysis) from the Glycolytic Rate Assay Kit. Subtraction of mitochondrial acidification from total PER (totalPER) results in glycolytic PER (glycoPER). n=5 for each group. **(D)** Quantification of glycolysis rate calculated from **(C)**. (Left) Level of basal glycolysis representing glycolysis rate in cells before injection of Rot/AA. (Middle) Percentage of glycoPER in totalPER before injection of Rot/AA. (Right) Levels of compensatory glycolysis representing glycolysis rate in cells following the addition of Rot/AA. **(G)** GSEA analysis of OXPHOS related genes in GM (negative) versus M (positive). **(H)** GM and M cells stained with Mito-Tracker Green to visualize the mitochondria in green and Hoechst 33342 to visualize the nucleus in cyan (left). Scale bars, 50 μm. (right) Quantification of mitochondrial fluorescence integrated density per cell. n=3 for each group. **(I)** Relative mtDNA content of GM and M cells measured by RT-PCR through the relative DNA content of mtDNA gene mt-Nd1 to nDNA gene β-actin. n=3 for each group. **(J)** Hierarchical cluster analysis for mitochondrial genes expression. Color scale represents scaled FPKM values normalized using the z-score approach. **(K)** OCR of GM and M when they were sequentially treated with oligomycin (inhibitors of ATP synthase), FCCP (uncoupling agent, collapsing the proton gradient and disrupting the mitochondrial membrane potential), and Rot/AA from the XF Cell Mito Stress Test Kit. n=5 for each group. **(L)** Quantification of mitochondrial respiration calculated from **(J)**. Basal respiration is calculated by the respiration before injection of oligomycin. ATP-production coupled respiration represents the respiration decrease upon injection of oligomycin. Maximal respiration is calculated by respiration after injection of FCCP and before injection of Rot/AA. Spare capacity is attained by the differences between maximal respiration and basal respiration. **(M)** Metabolic potential measured by Cell Energy Phenotype Test Kit. Metabolic potential is calculated by the percentage of OCR or ECAR under the stressed state in OCR or ECAR under the basal state. n=5 for each group. **(Q)** Diagram of glucose metabolism. Red and green color indicates gene or metabolite upregulated in M or GM, respectively, with cutoff |log_2_FC| > 0.25. * indicates |log_2_FC| > 0.25 and *P* < 0.05. For **(B, D, F, H, I, L, O, P)**, unpaired student’s t-test is performed, and for **(M)**, two-way ANOVA is performed. *P* < 0.05 is indicated by *.

Glucose metabolism (glycometabolism) is the major energy source to fuel cell functions (26); its associated functional terms were enriched in both GM and M samples (**Figures 1C, H**). We examined the major gene expression profile and relative amounts of intermediate metabolites of three major glycometabolic pathways: glycolysis, TCA cycle, and PPP, respectively (27). For glycolysis, half of the fourteen selected differentially expressed genes were upregulated in M and the remaining ones were upregulated in GM (**Figure 2A**). In detail, GM and M exhibit different preferences for enriching enzyme isozymes, such as Hk1 in M and Hk2 in GM, Pfkm in M and Pfkp and Pfkl in GM, and Eno2 in M and Eno1 in GM (**Figures 2A, Q**). The intermediate metabolites for the energy payoff stage, including 3-phospho-D-glycerate and phosphoenolpyruvate, were upregulated in M, while β-D-fructose-6-phosphate for the energy investment stage was upregulated in GM (**Figure 2B**), which indicates a possible higher preference for glycolysis in M cells and more abundant glucose storage (F6P) in GM cells (**Figure 2Q**). The intracellular glucose levels were determined using experimental assay, and, consistently, intracellular glucose storage is significantly higher in GM macrophages than their M counterparts (**Figure S2A**). In order to further determine which type of BMDMs have a more potent glycolysis capability, we conducted a glycolytic rate assay by Seahorse Extracellular Flux Analyzer, which is a real-time measurement to determine metabolic phenotypes of cells (28) (**Figure 2C**). No significant differences were observed regarding the absolute value of PER derived from glycolysis of M and GM at the steady state (basal glycolysis) (**Figure 2D**). However, the proportion of PER derived from glycolysis in total PER (including mitochondrial acidification derived PER) was significantly higher in M than in GM (**Figure 2D**), indicating a preference for anaerobic glycolysis and lactate production, rather than the continuation to TCA cycle in M. For GM cells, the absolute rate of compensatory glycolysis, the rate of glycolysis in cells following mitochondrial inhibitors’ addition to block OXPHOS, was significantly upregulated (**Figure 2D**), indicating that GM has a stronger capacity to fulfill anaerobic respiration under an anoxic environment.

Following glycolysis in aerobic respiration is the coupling of TCA cycle with OXPHOS (27). Most of the selected TCA cycle genes (8 out of 10) were upregulated in GM cells, including TCA cycle rate-limiting enzymes Idh3a and Ogdh (**Figure 2E**), and a relatively higher abundance of citrate was also detected in GM (**Figure 2F**). This is consistent with the weaker ability of M to proceed to TCA cycle under steady state as mentioned above. Thus, we concluded that anaerobic respiration is dominant in the cellular respiration of M, while aerobic respiration is dominant in that of GM. Next, we explored OXPHOS capacity of GM and M cells, the ultimate pathway of cellular respiration (29). GSEA analysis indicated that GM exhibited a strong upregulated transcriptional OXPHOS signature when compared to M (**Figure 2G**). Through a mitochondrial fluorescence tracker, we detected more intense mitochondrial signature in GM (**Figures 2H**, **S2B**). Consistently, higher mitochondrial DNA (mtDNA) copy number per cell, which represents the content of mitochondrial biogenesis, was significantly higher in GM cells (**Figure 2I**). Besides, mtDNA encodes 13 mitochondrial proteins as components of the electron transport chain (ETC) that are essential for OXPHOS and ATP synthesis (30, 31). As shown in **Figure 2J**, all 13 ETC genes were significantly upregulated in GM group. To further verify the high OXPHOS capacity in GM, seahorse cell mitochondrion stress test was utilized to detect mitochondrion respiration. Mitochondrial respiration modulators were sequentially added to reveal the key parameters of OXPHOS (**Figure 2K**). Our data showed that the respiration rate at steady state, ATP-production coupled respiration, maximal respiration under stress state, and spare respiration capacity were all significantly increased in GM (**Figure 2L**). Using seahorse cell energy phenotype test, we also found that GM exhibited a more intense energy-producing response in the stressed state, which meant that GM possessed a more energetic metabolic phenotype in both glycolysis and OXPHOS (**Figures 2M**, **S2C**).

As a metabolic pathway parallel to glycolysis, PPP provides NADPH for other metabolic pathways through oxidative phase, and also enables various carbohydrates to be converted into glycolytic intermediates through non-oxidative phase (32). In oxidative phase that begins at α-D-glucose 6-phosphate and ends in D-ribulose 5-phosphate (**Figure 2Q**), we found upregulated gene expressions of G6pdx and Pgls, and a higher abundance of 6-phospho-D-gluconate in M (**Figures 2N, O**), indicating the active PPP oxidative phase in M. In non-oxidative phase that starts from D-ribulose 5-phosphate to the end (**Figure 2Q**), we found increased Rpia gene expression, and higher abundances of D-ribose 5-phosphate, D-erythrose 4-phosphate (**Figure 2P**), and β-D-fructose 6-phosphate (intermediate substrate for glycolysis) (**Figure 2B**) in GM, indicating the active PPP non-oxidative phase in GM.

Hence, these results demonstrate that M macrophages depend much more on glycolysis and possess active PPP oxidative phases, while GM macrophages exhibit a high intensity of glucose oxidation, mitochondrial respiratory function, and PPP non-oxidative process (**Figure 2Q**).

### 3.3 Enriched fatty acid synthesis, bile acid synthesis and lipid accumulation in M

**Figure 3:**
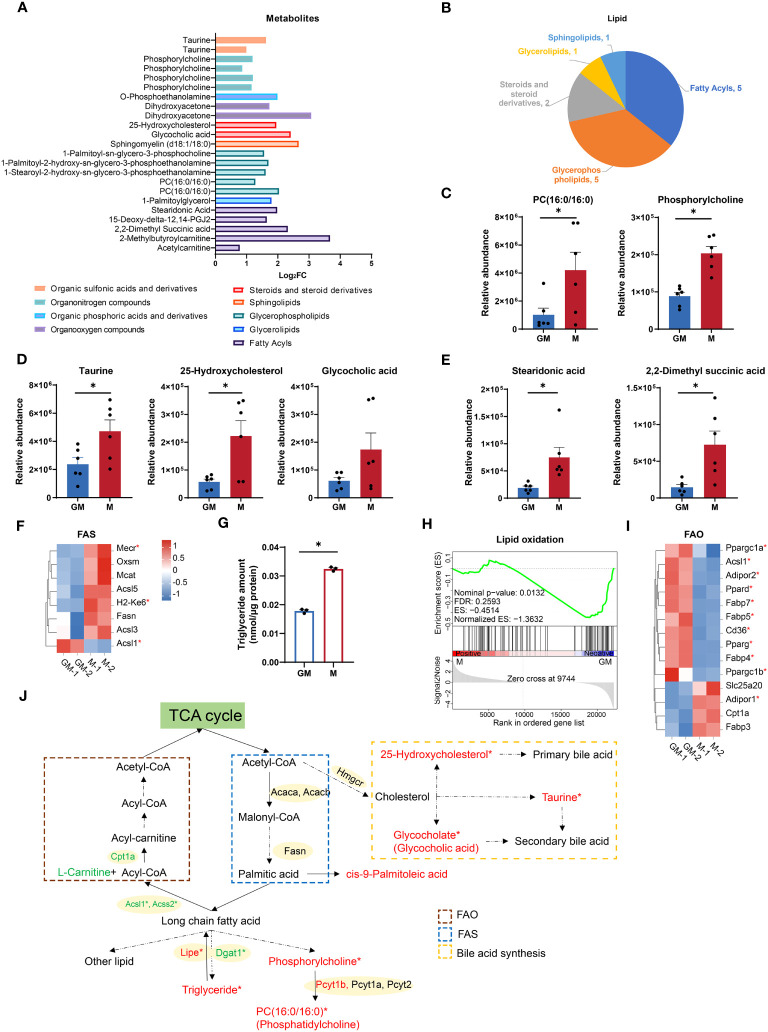
Comparison of lipid metabolism between GM and M. **(A)** Intergroup distribution of intermediate metabolites of different types of lipid metabolism. The x-axis represents the log_2_FC values of M/GM. **(B)** The metabolites class distribution of differential metabolites related to lipid metabolism in the comparison of GM and M groups. **(C-E)** Relative abundance of lipid metabolic metabolites measured by LC-MS. n=6 for each group. **(F, I)** Hierarchical cluster analysis for the gene expression of FAS (KEGG database) **(F)** and FAO **(I)**. Color scale represents scaled FPKM values normalized using z-score approach. These genes were chosen from complete genes list by cutoff |log_2_FC| > 0.25. * indicates *P* < 0.05. **(G)** Triglyceride concentration of GM and M measured by Adipogenesis Colorimetric/Fluorometric Assay Kit. n=6 for each group. **(H)** GSEA analysis of lipid oxidation related gene in GM (negative) versus M (positive). **(J)** Diagram of lipid metabolism. Red and green color indicates gene or metabolite is upregulated in M or GM, respectively, with |log_2_FC| > 0.25. * indicates |log_2_FC| > 0.25 and *P* < 0.05. For **(C, D, E, G)**, unpaired student’s t-test is performed and *P* < 0.05 is indicated by *.

Lipid metabolism, in addition to glucose metabolism, is crucial for energy storage and supplies for biological functions through the decomposition and synthesis of lipids (33). According to our metabolic data, numerous lipids and metabolites involved in lipid metabolism were significantly upregulated in M samples (**Figures 3A, B**). For example, metabolites for glycerophospholipid metabolism (PC(16:0/16:0) and phosphorylcholine) and those for primary bile acid biosynthesis (glycocholic acid, 25-hydroxycholesterol and taurine) were both highly enriched in M group (**Figures 3C, D**). Fatty acids are the major components of various lipids and play a key role in lipid metabolism as metabolic fuel (34). Fatty acids such as stearidonic acid and 2,2-dimethyl succinic acid were also increased in M group (**Figure 3E**). Fatty acid synthesis utilizes NADPH as a reducing agent (35). Additionally, as the major source of NADPH, PPP oxidative phase is more active in M cells (**Figure 2O**). The majority of selected FAS associated genes (Mmu00061in KEGG Pathway), such as Mecr and H2-Ke6, were higher in M, while upregulated Acsl1 in GM could provoke fatty acid to perform β-oxidation (**Figure 3F**) (36). Consequently in M, we found a larger abundance of triglyceride, as which fatty acids are stored in cells (**Figure 3G**) (37). Therefore, M macrophages are more active in FAS, primary bile acid synthesis, and lipid storage than GM macrophages.

As a major energy source, energy production by fatty acids, mainly by oxidation, is second only to glucose (38). Next, we explored FAO capacities of both kinds of macrophages. The GSEA analysis showed that M exhibited downregulated gene expressions in lipid oxidation (**Figure 3H**). In addition, all the selected genes for FAO with significance (*P* < 0.05) were upregulated in GM (**Figure 3I**) except for Adipor1. Considering that acetyl-CoA, the end product of FAO, would enter the TCA cycle for ATP production (39), the strong FAO signature of GM is consistent with its higher TCA cycle level, OXPHOS capacity, and mitochondrial contents. These data indicated that M macrophages are burdened with higher fat storage, while GM macrophages possess a stronger ability to oxidize lipid and provide energy for cells (**Figure 3J**).

### 3.4 The enhanced arginine-to-ornithine/urea metabolic cascade in GM

**Figure 4:**
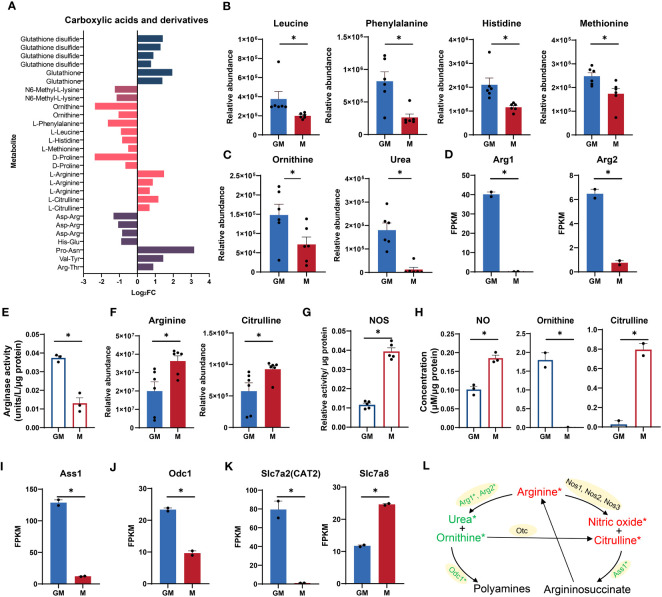
Comparison of arginine metabolism between GM and M. **(A)** Intergroup distribution of carboxylic acids and derivatives. The x-axis represents the log_2_FC values of M/GM. **(B, C, F)** Relative abundance of essential amino acids **(B)** and arginine metabolic metabolites **(C, F)** measured by LC-MS. n=6 for each group. **(D, I, J, K)** FPKM values of Arg1 and Arg2 **(D)**, Ass1 **(I)**, Odc1 **(J)**, Slc7a2, and Slc7a8 **(K)** in our RNA-seq data. n=2 for each group. **(E)** Arginase activity of GM and M measured by Arginase Activity Assay Kit. n=3 for each group. **(G)** NOS relative activity of GM and M measured by Nitric Oxide Synthase Assay Kit. n=5 for each group. **(H)** NO concentration (left) in culture media of GM and M measured by Nitric Oxide Assay Kit. n=3 for each group. Ornithine (middle) and citrulline (right) concentration in culture media of GM and M measured by Ornithine and Citrulline Fluorometric Assay Kit. n=2 for each group. **(L)** Diagram of arginine metabolism. Red and green color represents gene or metabolite upregulated in M or GM groups, respectively, with |log_2_FC| > 0.25. * indicates |log_2_FC| > 0.25 and *P* < 0.05. For **(B–K)**, unpaired student’s t-test is performed and *P* < 0.05 is indicated by *.

According to our RNA-seq and metabolic data, amino acid metabolic pathways, especially arginine metabolism were highly enriched in GM samples (**Figures 1D, H**). As shown in **Figure 4A**, there was a panel of carboxylic acids and derivatives which were significantly regulated in both types of macrophages. The essential amino acids such as leucine, phenylalanine, histidine, and methionine were significantly upregulated in GM, pointing to frequent amino acid uptakes and protein synthesis (**Figures 4A, B**).

As a conditionally essential amino acid for mammals, arginine is mainly metabolized by nitric oxide synthase (NOS) and Arg to produce citrulline and NO, and ornithine and urea, respectively (40, 41) (**Figure 4L**). The ornithine level and urea production were significantly higher in GM samples (**Figure 4C**). The expression levels of the indicated enzyme genes Arg1 and Arg2 were also significantly increased (**Figure 4D**). Consistently, the enzyme activity of Arg is significantly higher in GM samples (**Figure 4E**). For M cells, the arginine level and production of citrulline were significantly higher (**Figure 4F**). The indicated enzyme genes including Nos1, Nos2, and Nos3 were all upregulated in M but with low expression levels and no significant difference (data not shown). Nevertheless, the enzyme activity of NOS was significantly upregulated in M macrophages, accounting for their more active citrulline production from arginine (**Figure 4G**). The NO production accompanied by citrulline synthesis from arginine is determined using NO assay, with M macrophages releasing more NO in cell supernatant (**Figure 4H**). The preference of ornithine production for GM and citrulline production for M was also verified using experimental assays in cell supernatant (**Figure 4H**). The argininosuccinate synthetase 1 (Ass1), the enzyme responsible for arginine biosynthetic pathway (42) (**Figure 4L**), was significantly upregulated in GM (**Figure 4I**). Besides, the ornithine decarboxylase (ODC), encoded by Odc1, a rate-limiting enzyme in the process of metabolizing to polyamines involved in a multitude of cellular processes including cell development, oxidative DNA damage, and differentiation (43), was transcriptionally upregulated in GM (**Figures 4J, L**). We also determined the gene expression levels of arginine transporters in our RNA-seq data. As shown in **Figure 4K**, the canonical arginine transporter Slc7a2 (CAT2) (44) was uniquely expressed in GM cells, while transporter Slc7a8 (45) was significantly upregulated in M cells, indicating the differential preference for arginine transport in these two types of macrophages. Collectively, GM macrophages exhibit stronger arginine-to-ornithine/urea pathway activity while M macrophages are more active in the arginine to NO/citrulline cascade (**Figure 4L**).

### 3.5 High consistency of RNA-seq data of mouse BMDMs with proteomic data of mouse BMDMs and RNA-seq data of human MDMs

**Figure 5:**
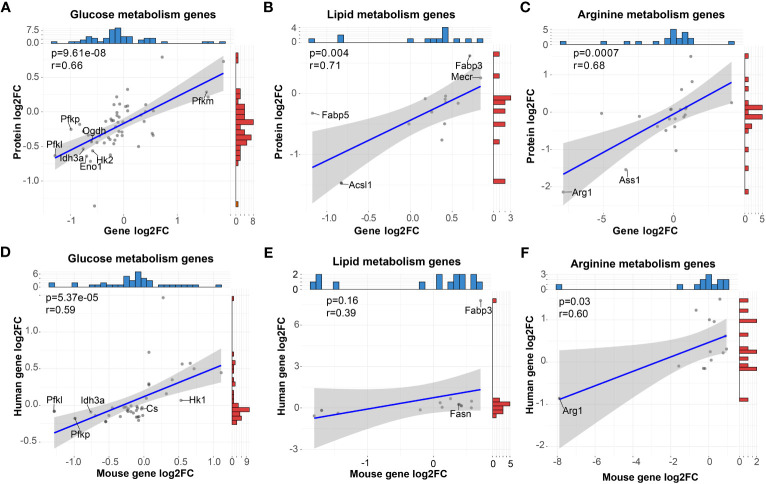
High correlation of metabolic gene expression between mRNA and protein of mouse BMDMs and mRNA of human MDMs *in vitro.* **(A–C)** Pearson correlation analysis of mRNA and protein levels of genes (dots) related to glucose **(A)**, lipid **(B)** and arginine **(C)** metabolism. **(D–F)** Pearson correlation analysis of mRNA levels of genes (dots) related to glucose **(D)**, lipid **(E)**, and arginine **(F)** metabolism derived from mouse BMDMs and human MDMs. For **(A–F)**, blue and red histograms represent the data distribution of the x-axis and y-axis, respectively.

To determine the consistency of the metabolism characteristics at protein level and RNA level, we performed correlation analyses of our RNA-seq data with our proteomic data of GM and M from the iProX database (PXD041180, http://proteomecentral.proteomexchange.org/cgi/GetDataset?ID=PXD041180). As shown in **Figures 5A–C**, significantly positive correlation of mRNA and protein levels of glucose, lipid and arginine metabolism (P < 0.05) were observed. For instance, Pfkm for glucose metabolism were upregulated in M while Acsl1 and Fabp5 for lipid metabolism and Arg1 and Ass1 for arginine metabolism were downregulated in M. As a result, in terms of metabolism, the transcriptome is compatible with the protein level.

To explore the consistency of mouse macrophages with human macrophages both primed by GM-CSF and M-CSF, we analyzed the RNA-seq data of human MDMs from the public GEO database (GSE135491). Data were normalized, and comparison analysis between GM-MDMs and M-MDMs were conducted. For mouse genes involved in the above metabolism analysis, we sorted out the orthologous human genes and correlated their expression with mouse RNA-seq data (**Figures 5D–F**). High positive correlations (*P* < 0.05) were observed between these two species in terms of glucose and arginine metabolism, while positive correlation with less significance (*P* = 0.16) was observed for lipid metabolism, including consistent gene expression trends of Pfkl, Pfkp, Idh3a, Cs, Hk1, Fabp3, Fasn, and Arg1. These results support a metabolic compatibility of *in vitro* GM-CSF and M-CSF primed mouse macrophage models with the human body to some extent.

### 3.6 Potential connection between identified metabolic gene expression and canonical inflammatory and phagocytotic functions

**Figure 6:**
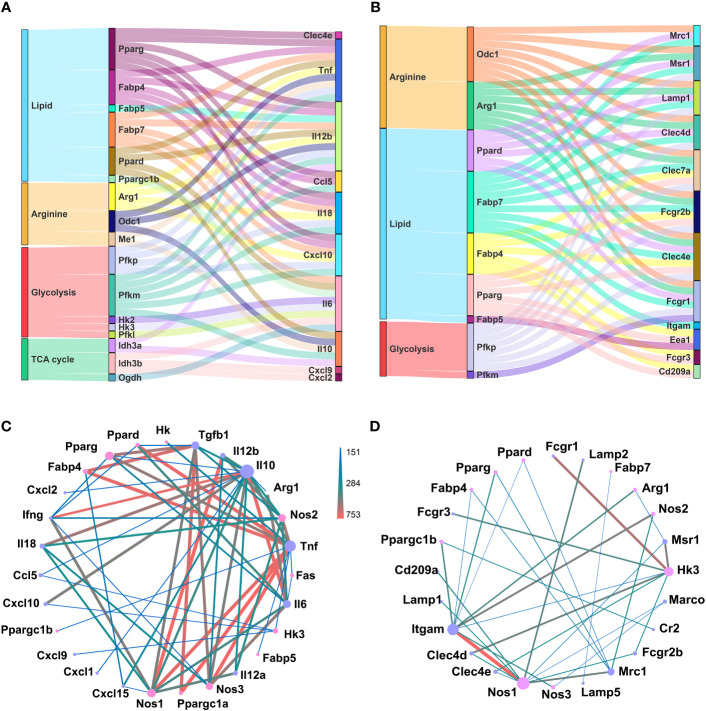
Potential connection between identified metabolic genes and function of inflammation and phagocytosis of macrophages. **(A, B)** Top 50 pairs of metabolic genes (left column) and chemokine and cytokine genes (right column) **(A)** and phagocytosis genes (right column) **(B)** with the highest correlation. Each line connects a pair of correlated genes. **(C, D)** PPI analysis of selected metabolic genes (pink nodes) and chemokine and cytokine genes (purple nodes) **(C)** and phagocytotic genes (purple nodes) **(D)**. The node size represents the connectivity degree. Lager nodes, larger connectivity degree. The line thickness and color represent the combine score.

Macrophages could sense and immediately respond to invading pathogens, via chemokine and cytokine production and phagocytosis, partially through metabolism reprograming (46). Similarly, different differentiated status of GM and M regarding inflammatory factor release and phagocytosis could also be correlated with their metabolism preferences. We performed correlation analysis of metabolism genes’ expressions with those of cytokine and chemokine genes (**Figure S3**) and phagocytosis related genes (**Figure S4**), respectively, and sorted out the top 50 most correlated gene pairs (**Figures 6A, B**; **Tables S1, 2**). For chemokine and cytokine release, lipid metabolism and glycolysis were dominant, and among them, glycolysis gene Pfkm exhibited a positive correlation, while lipid metabolic genes Fabp4 and Fabp7 showed a negative correlation with Tnf, Il18, and Cxcl10 (**Figure 6A**; **Table S1**). For phagocytosis, lipid and arginine metabolic pathways were dominant. Arginine metabolic genes Arg1 and Odc1, and lipid metabolic genes Fabp7 were positively related with Clec7a, Fcgr2b, and Msr1, while being negatively related with Clec4d, Clec4e, Fcgr1, and Lamp1 (**Figure 6B**; **Table S2**). Protein-protein interaction (PPI) analysis of these genes was also performed (**Figures 6C, D**). For chemokine and cytokine release, lipid metabolic genes Pparg, Ppargc1a, and Fabp4 intensely interacted with Tgfb1 and Tnf, and arginine metabolic gene Nos1, Nos2, and Nos3 intensely interacted with Il12b, Tnf, Il10, Il18, and Tgfb1 (**Figure 6C**). For phagocytosis, arginine metabolic gene Nos1 is strongly connected with Itgam and Lamp2, and glycolysis gene Hk3 is strongly connected with Fcgr1, Fcgr3, Msr1, and Clec4d (**Figure 6D**). All these data described the potential tight connections between metabolism and the typical inflammatory and phagocytic actions of macrophages.

## 4 Discussion

Macrophages undergo maturation and activation to fulfill their specific functional roles both *in vitro* and *in vivo*, which is mainly mediated by various stimuli or the combinations of the stimuli, including growth factors, cytokines, metabolites, and microbial ligands (2, 47). Currently, there are two sets of *in vitro* BMDM models that are well-established and widely used in the studies: GM-CSF and M-CSF primed macrophage differentiation/maturation model (8, 9), and LPS/IFNγ (M1) and IL4/IL13 (M2) stimulated macrophage polarization which usually follows M-CSF-induced maturation (2, 48). GM-CSF primed BMDMs are referred to as M1-like macrophages with a “proinflammatory” cytokine profile and some overlapping features with LPS/IFNγ-induced M1, while M-CSF primed ones are referred to as M2-like macrophages with an “anti-inflammatory” cytokine profile and some features of IL4/IL13-induced M2 (2, 8, 49). Although similarity exists, both the detailed inflammatory status (2) and the metabolic characteristics regarding the preference of cytoplasmic glycolysis and mitochondrial TCA cycle, OXPHOS, FAO, and arginine metabolism differed from each other based on previously published literatures and the current study (12, 13, 50). For example, in LPS/IFNγ-induced macrophages, arginine is converted to NO by iNOS, and in IL4-induced macrophages, arginine is metabolized to urea and ornithine by Arg1 (41). However, in the present study, GM macrophages were inclined towards the arginine-to-urea/ornithine cascade, which was driven by Arg1 and Arg2, whereas M macrophages tended towards the arginine-to-NO/citrulline pathway and exhibited higher NOS activity. In contrast to their high expression in LPS/IFNγ-polarized classically activated M1 macrophages, the gene expression levels of NOS family members were at low levels in both GM and M cells and no significant differences were observed. According to our previously published RNA-seq data (6), iNOS is a strong LPS-induced gene regardless of the differentiation/maturation with M-CSF or GM-CSF (FPKM value 0.17 vs 226.75 for M vs M_LPS_3h; FPKM value 0.15 vs 79.69 for GM vs GM_LPS_3h). In addition, LPS induced iNOS expression catalyzes the massive production of NO and consequently impaired the OXPHOS activity of macrophages (51, 52). Similarly, IL4 treatment can substantially induce the expression of Arg1 (53), which is consistent with the catalysis of arginine to urea and ornithine in alternative IL4-activated M2 macrophages. Therefore, the metabolic status of macrophages was highly plastic, able to rapidly adapt to their local microenvironment, which may contain different stimuli or combinations of stimuli, to tune their immunometabolic status and perform the different functions required to respond to these stimuli.

**Figure 7:**
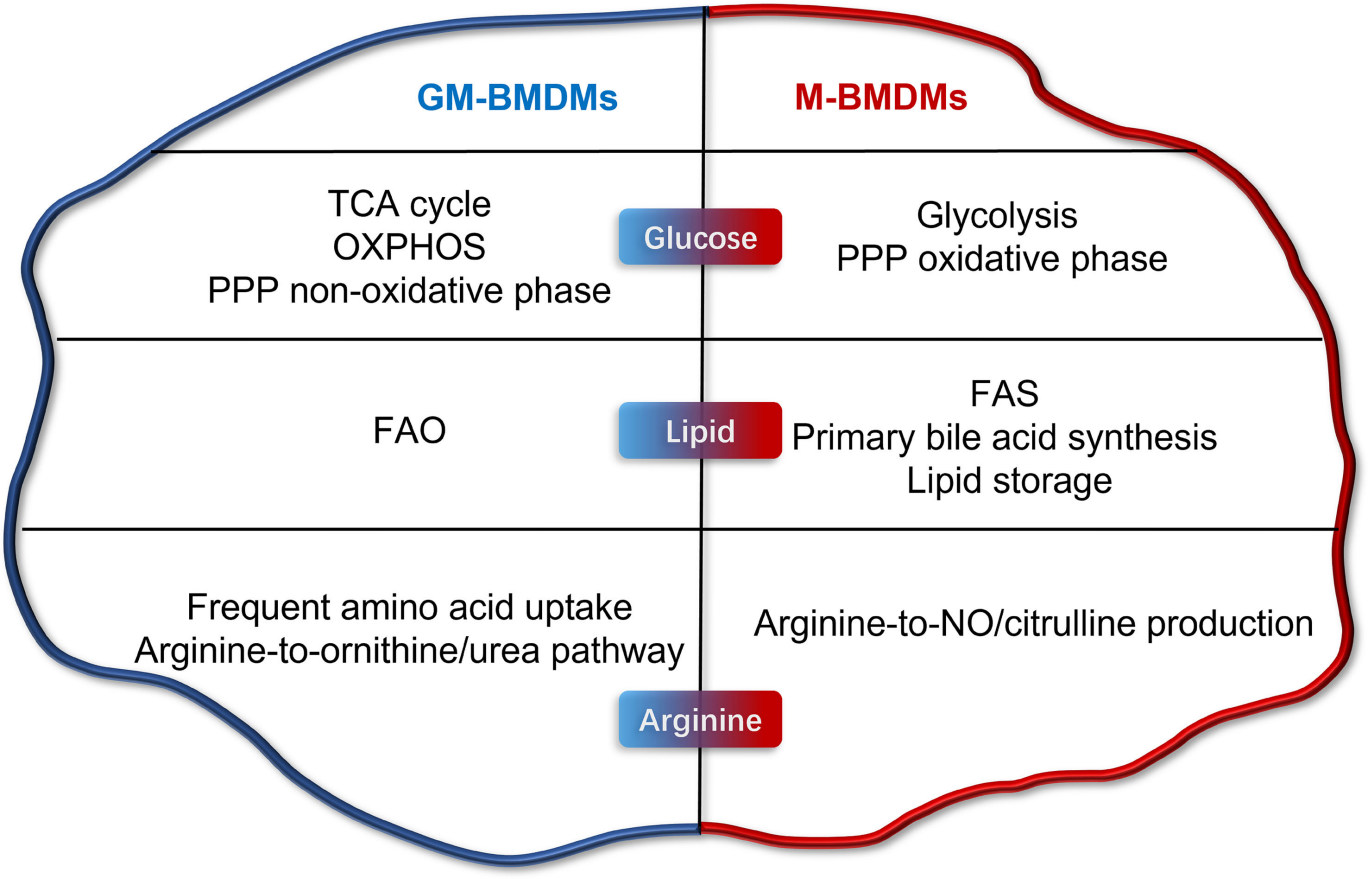
Metabolic programming of GM-CSF- and M-CSF- differentiated mouse macrophages *in vitro*. Briefly, GM cells present higher level of TCA cycle, OXPHOS, PPP non-oxidative phase, FAO, frequent amino acid uptake, and arginine-to-ornithine/urea pathway. M cells possess higher levels of glycolysis, FAS, primary bile acid synthesis, lipid storage, and arginine-to-NO/citrulline pathway production.

Several other studies have attempted to elucidate the influences of GM-CSF and M-CSF (or L929-CM) on the metabolic status of macrophages (16–18). Yi Rang Na et al. investigated the metabolic status of GM-CSF and L929-CM primed mouse BMDMs by proteomic analysis (16). Consistent with the current study, the maximum glycolytic capacity (complementary glycolytic capacity) was found to be higher in GM-BMDMs. However, L929-CM and M-CSF-primed BMDMs were reported to be metabolically different to some extent (19). Numerous M-BMDM-enriched genes involved in glucose, lipid and amino acid metabolism in our study were not identified in L929-CM-derived macrophages. Elena Izquierdo et al. reported the glucose metabolic characteristics of GM-CSF and M-CSF-primed human MDMs (hMDMs) (18). The high mitochondrial ATP production and glycolysis capacity were shared by mouse GM-BMDMs (mGM-BMDMs) and hGM-MDMs; however, the basal glycolysis of GM and M was not consistent in human and mouse macrophages. The glycolytic gene Pfkp was both upregulated and Pfkfb2 was both downregulated in mGM-BMDMs and hGM-MDMs, while the glycolytic metabolite phosphoenolpyruvate (PEP) was increased in hGM-MDMs but decreased in mGM-BMDMs. In 2017, Sina Tavakoli et al. investigated the influences of GM-CSF and M-CSF on the metabolism of mouse peritoneal macrophages (17). Consistent with the present study, M-CSF primed peritoneal macrophages tend to undergo glycolysis at steady state. However, a panel of glycolytic genes such as Hk2, Pfkp, Pgam1, and Eno1 were not consistent with the present study. Due to the differences in species, macrophage sources, culture conditions, growth factor sources, and treatment time, there are consistencies and inconsistencies in the conclusions of GM-CSF/M-CSF regulated metabolic changes of macrophages *in vitro*. In the current study, we profiled the metabolic landscape of GM- and M- BMDMs, and illustrated their metabolic preferences and in glucose, lipid, and arginine metabolism using RNA-seq and metabolomics (**Figure 7**). Furthermore, high consistency in metabolic gene expression was observed between mouse macrophage mRNA and protein levels, and human macrophage in mRNA levels. The potential connection of these metabolic pathways with inflammation and phagocytosis was also discussed, laying the groundwork for mechanistic elucidation.

The present study also reveals an intriguing phenomenon that warrants further investigation: as a member of innate immunity, GM macrophages rely heavily on aerobic respiration, and yet respond rapidly to the stressed environment (compensatory glycolysis). Aerobic respiration is up to 16 times more efficient than anaerobic respiration (about 32 vs 2 ATP production per glucose) (54). They share the initial glycolytic pathway, but aerobic metabolism continues with the TCA cycle and OXPHOS, which occurs in the mitochondria of eukaryotic cells (55). In our data, almost all the TCA cycle related genes (from pyruvate to acetyl-CoA to TCA cycle) were significantly upregulated in GM macrophages, as well as 13 mitochondrial encoded ETC components. The strong mitochondrial respiratory capacity allows GM cells to fully utilize acetyl-CoA, derived from glycolysis and lipid oxidation, for energy production under aerobic conditions. Meanwhile, abundant glucose and fructose-6P were accumulated in GM macrophages, and Glut1 (Slc2a1) for glucose transport and Hk2 for glucose to glucose-6P were both significantly upregulated in the GM group to provide additional glucose and fructose-6P for anaerobic glycolysis. In addition, the key components for glycolysis such as Pfkp, Aldoc, Pgam1 and Eno1 were preferentially highly expressed in GM cells. All of these gene expression and metabolite accumulation provide GM cells with flexible glycolytic capacities in response to an anaerobic environment. The high flexibility of the ATP production pathway allows the GM cells to respond rapidly to the environment and ensure a sufficient energy supply. This phenomenon may explain its prolific production of inflammatory chemokines and cytokines in response to pathogen infection such as LPS (49) and its remarkable effects on tumor elimination (unpublished data). Nevertheless, further research is needed to determine the underlying mechanism by which GM cells possess enhanced mitochondrial function and how this affects their immune response.

Beyond the aspect of cell respiration, in the result of potential connection between metabolic genes and phagocytotic functions, Lamp1, a member of lysosome-associated membrane glycoproteins involving in autophagy process (56), and *Clec* genes (Clec7a, Clec4d, and Clec4e), which belongs to C-type lectin receptors participating in pathogens clearance (57), stand out in our data. Autophagy is a pathway by which macrophages eliminate pathogens, and the autophagy flux is closely related with cellular metabolism (58, 59). Arginine metabolism genes Arg1 and Odc1 and FAO genes Fabp7, which were all dominantly expressed in GM, positively correlated with Clec7a which highly expressed in GM (**Figure 6B**; **Table S2**). According to the research conducted by Wang et al., Clec7a+ M1 macrophages exhibited potent pro-inflammatory and phagocytic effects compare to Clec7a- population (60). Once pathogens infiltrate cells, their eradication occurs by the lysosome through a series of gene regulation and protein interaction, such as Lamp1, P62, and LC3 in the autophagy process (61, 62). By integrating these findings and our results, the production of polyamines, mediated by Arg1 and Odc1, alongside the fatty acid breakdown process, mediated by Fabp7 and other FAO genes, exert potentially positive effect on pathogenic phagocyte and intracellular pathogen autophagy. Polyamines regulate the translation factor eIF5A, ultimately promoting mitochondrial gene expression and OXPHOS capacity in macrophages (63). Moreover, the FAO pathway provides reducing equivalents and then induces OXPHOS (64). During quiescence, OXPHOS generate low-level of ROS that induce autophagy via attenuation of ATG4B activity (65). These theories hold promise in explicating the potential role of polyamines and fatty acid breakdown process in intracellular pathogen autophagy mechanism, although further robust experimentation is warranted for their validation.

There are several limitations in this study. Firstly, GM-CSF differentiated cells encompass a heterogeneous population comprising both CD11c+ MHC II+ macrophages and dendritic cells (10). Currently, it remains unclear to which extent the specific cell subpopulation contributes to the distinctive metabolic pathways of GM-BMDMs. Secondly, in addition to GM-CSF and M-CSF, macrophages *in vivo* are influenced by a complex array of other factors (66). The *in vitro* macrophage model does not entirely capture the intricate metabolic alterations induced by the *in vivo* environment. Further more sophisticated studies using purified cell populations or even at single cell levels will better resolve these limitations.

In conclusion, we performed systematic metabolic profile of GM-CSF and M-CSF differentiated macrophages *in vitro*. The multi-omics data, coupled with experimental validation, serve as a rich resource for deep understanding of immunometabolism of macrophages and pave the way for finding targets by focusing on the feature metabolic pathways of macrophages.

## Data availability statement

The datasets presented in this study can be found in online repositories. The names of the repository/repositories and accession number(s) can be found in the article/**Supplementary Material**.

## Ethics statement

Ethical approval was not required for the studies involving humans because we utilize RNA-seq data of human MDMs from public database (GEO: GSE135491). The studies were conducted in accordance with the local legislation and institutional requirements. The human samples used in this study were acquired from public database (GEO: GSE135491). Written informed consent to participate in this study was not required from the participants or the participants’ legal guardians/next of kin in accordance with the national legislation and the institutional requirements. The animal study was approved by the Committee of Experimental Animals of School of Medicine and Pharmacy, Ocean University of China. The study was conducted in accordance with the local legislation and institutional requirements.

## Author contributions

All authors participated in interpreting the whole data. CZ and JY supervised the study; QZ, QS, SL, YX, DG, YL, GZ, and LW performed and evaluated individual experiments; QZ and QS performed bioinformatical analyses; QZ, QS, and PL wrote the manuscript with the contributions from all the authors. All authors contributed to the article and approved the submitted version.

## References

[1] Gordon S Taylor PR. Monocyte and macrophage heterogeneity. Nat Rev Immunol (2005) 5(12):953–64. doi: 10.1038/nri1733 16322748

[2] Murray PJ Allen JE Biswas SK Fisher EA Gilroy DW Goerdt S. Macrophage activation and polarization: Nomenclature and experimental guidelines. Immunity (2014) 41(1):14–20. doi: 10.1016/j.immuni.2014.06.008 25035950 PMC4123412

[3] Ushach I Zlotnik A. Biological role of granulocyte macrophage colony-stimulating factor (gm-csf) and macrophage colony-stimulating factor (m-csf) on cells of the myeloid lineage. J Leukoc Biol (2016) 100(3):481–9. doi: 10.1189/jlb.3RU0316-144R PMC4982611 27354413

[4] Wei W Zhang Y Song Q Zhang Q Zhang X Liu X. Transmissible er stress between macrophages and tumor cells configures tumor microenvironment. Cell Mol Life Sci (2022) 79(8):403. doi: 10.1007/s00018-022-04413-z 35799071 PMC11073001

[5] Zhao Z Zhang Y Gao D Zhang Y Han W Xu X. Inhibition of histone h3 lysine-27 demethylase activity relieves rheumatoid arthritis symptoms *via* repression of il6 transcription in macrophages. Front Immunol (2022) 13:818070. doi: 10.3389/fimmu.2022.818070 35371061 PMC8965057

[6] Song Q Zhang Y Zhou M Xu Y Zhang Q Wu L. The culture dish surface influences the phenotype and dissociation strategy in distinct mouse macrophage populations. Front Immunol (2022) 13:920232. doi: 10.3389/fimmu.2022.920232 35874686 PMC9299442

[7] Zhang L Pavicic PG Datta S Song Q Xu X Wei W. Unfolded protein response differentially regulates tlr4-induced cytokine expression in distinct macrophage populations. Front Immunol (2019) 10:1390. doi: 10.3389/fimmu.2019.01390 31293572 PMC6598306

[8] Fleetwood AJ Dinh H Cook AD Hertzog PJ Hamilton JA. Gm-csf- and m-csf-dependent macrophage phenotypes display differential dependence on type i interferon signaling. J Leukoc Biol (2009) 86(2):411–21. doi: 10.1189/jlb.1108702 19406830

[9] Lacey DC Achuthan A Fleetwood AJ Dinh H Roiniotis J Scholz GM. Defining gm-csf- and macrophage-csf-dependent macrophage responses by in *vitro* models. J Immunol (2012) 188(11):5752–65. doi: 10.4049/jimmunol.1103426 22547697

[10] Helft J Böttcher J Chakravarty P Zelenay S Huotari J Schraml BU. Gm-csf mouse bone marrow cultures comprise a heterogeneous population of cd11c(+)mhcii(+) macrophages and dendritic cells. Immunity (2015) 42(6):1197–211. doi: 10.1016/j.immuni.2015.05.018 26084029

[11] Pearce EL Pearce EJ. Metabolic pathways in immune cell activation and quiescence. Immunity (2013) 38(4):633–43. doi: 10.1016/j.immuni.2013.04.005 PMC3654249 23601682

[12] Liu Y Xu R Gu H Zhang E Qu J Cao W. Metabolic reprogramming in macrophage responses. biomark Res (2021) 9(1):1. doi: 10.1186/s40364-020-00251-y 33407885 PMC7786975

[13] Van den Bossche J O'Neill LA Menon D. Macrophage immunometabolism: Where are we (going)? Trends Immunol (2017) 38(6):395–406. doi: 10.1016/j.it.2017.03.001 28396078

[14] Koo S-J Garg NJ. Metabolic programming of macrophage functions and pathogens control. Redox Biol (2019) 24:101198. doi: 10.1016/j.redox.2019.101198 31048245 PMC6488820

[15] Puthenveetil A Dubey S. Metabolic reprograming of tumor-associated macrophages. Ann Transl Med (2020) 8(16):1030. doi: 10.21037/atm-20-2037 32953830 PMC7475460

[16] Na YR Hong JH Lee MY Jung JH Jung D Kim YW. Proteomic analysis reveals distinct metabolic differences between granulocyte-macrophage colony stimulating factor (gm-csf) and macrophage colony stimulating factor (m-csf) grown macrophages derived from murine bone marrow cells. Mol Cell Proteomics (2015) 14(10):2722–32. doi: 10.1074/mcp.M115.048744 PMC4597147 26229149

[17] Tavakoli S Short JD Downs K Nguyen HN Lai Y Zhang W. Differential regulation of macrophage glucose metabolism by macrophage colony-stimulating factor and granulocyte-macrophage colony-stimulating factor: Implications for 18f fdg pet imaging of vessel wall inflammation. Radiology (2017) 283(1):87–97. doi: 10.1148/radiol.2016160839 27849433 PMC5375627

[18] Izquierdo E Cuevas VD Fernández-Arroyo S Riera-Borrull M Orta-Zavalza E Joven J. Reshaping of human macrophage polarization through modulation of glucose catabolic pathways. J Immunol (2015) 195(5):2442–51. doi: 10.4049/jimmunol.1403045 26209622

[19] de Brito Monteiro L Davanzo GG de Aguiar CF Corrêa da Silva F de Andrade JR Campos Codo A. M-csf- and l929-derived macrophages present distinct metabolic profiles with similar inflammatory outcomes. Immunobiology (2020) 225(3):151935. doi: 10.1016/j.imbio.2020.151935 32201093

[20] Kim D Langmead B Salzberg SL. Hisat: A fast spliced aligner with low memory requirements. Nat Methods (2015) 12(4):357–60. doi: 10.1038/nmeth.3317 PMC4655817 25751142

[21] Kanehisa M Furumichi M Tanabe M Sato Y Morishima K. Kegg: New perspectives on genomes, pathways, diseases and drugs. Nucleic Acids Res (2017) 45(D1):D353–D61. doi: 10.1093/nar/gkw1092 PMC5210567 27899662

[22] Subramanian A Tamayo P Mootha VK Mukherjee S Ebert BL Gillette MA. Gene set enrichment analysis: A knowledge-based approach for interpreting genome-wide expression profiles. Proc Natl Acad Sci U.S.A. (2005) 102(43):15545–50. doi: 10.1073/pnas.0506580102 PMC1239896 16199517

[23] Szklarczyk D Franceschini A Wyder S Forslund K Heller D Huerta-Cepas J. String v10: Protein-protein interaction networks, integrated over the tree of life. Nucleic Acids Res (2015) 43(Database issue):D447–D52. doi: 10.1093/nar/gku1003 PMC4383874 25352553

[24] Krämer A Green J Pollard J Tugendreich S. Causal analysis approaches in ingenuity pathway analysis. Bioinformatics (2014) 30(4):523–30. doi: 10.1093/bioinformatics/btt703 PMC3928520 24336805

[25] Leuthner TC Hartman JH Ryde IT Meyer JN. Pcr-based determination of mitochondrial DNA copy number in multiple species. Methods Mol Biol (2021) 2310:91–111. doi: 10.1007/978-1-0716-1433-4_8 34096001

[26] Mulukutla BC Yongky A Le T Mashek DG Hu W-S. Regulation of glucose metabolism - a perspective from cell bioprocessing. Trends Biotechnol (2016) 34(8):638–51. doi: 10.1016/j.tibtech.2016.04.012 27265890

[27] Lehninger AL Nelson DL Cox MM. Lehninger principles of biochemistry. (New York: W.H. Freeman) (2021).

[28] van der Windt GJW Chang C-H Pearce EL. Measuring bioenergetics in t cells using a seahorse extracellular flux analyzer. Curr Protoc Immunol (2016) 113:3.16B.1–3.16B.14. doi: 10.1002/0471142735.im0316bs113 PMC4864360 27038461

[29] Nolfi-Donegan D Braganza A Shiva S. Mitochondrial electron transport chain: Oxidative phosphorylation, oxidant production, and methods of measurement. Redox Biol (2020) 37:101674. doi: 10.1016/j.redox.2020.101674 32811789 PMC7767752

[30] Reznik E Wang Q La K Schultz N Sander C. Mitochondrial respiratory gene expression is suppressed in many cancers. Elife (2017) 6:e21592. doi: 10.7554/eLife.21592 28099114 PMC5243113

[31] Seidel-Rogol BL Shadel GS. Modulation of mitochondrial transcription in response to mtdna depletion and repletion in hela cells. Nucleic Acids Res (2002) 30(9):1929–34. doi: 10.1093/nar/30.9.1929 PMC113853 11972329

[32] Patra KC Hay N. The pentose phosphate pathway and cancer. Trends Biochem Sci (2014) 39(8):347–54. doi: 10.1016/j.tibs.2014.06.005 PMC4329227 25037503

[33] Yoon H Shaw JL Haigis MC Greka A. Lipid metabolism in sickness and in health: Emerging regulators of lipotoxicity. Mol Cell (2021) 81(18):3708–30. doi: 10.1016/j.molcel.2021.08.027 PMC8620413 34547235

[34] Rustan AC Drevon CA. Fatty acids: Structures and properties. Els (2005). doi: 10.1038/npg.els.0003894

[35] Schroeder B Vander Steen T Espinoza I Venkatapoorna CMK Hu Z Silva FM. Fatty acid synthase (fasn) regulates the mitochondrial priming of cancer cells. Cell Death Dis (2021) 12(11):977. doi: 10.1038/s41419-021-04262-x 34675185 PMC8531299

[36] Huh JY Reilly SM Abu-Odeh M Murphy AN Mahata SK Zhang J. Tank-binding kinase 1 regulates the localization of acyl-coa synthetase acsl1 to control hepatic fatty acid oxidation. Cell Metab (2020) 32(6):1012–27. doi: 10.1016/j.cmet.2020.10.010 PMC7710607 33152322

[37] Alves-Bezerra M Cohen DE. Triglyceride metabolism in the liver. Compr Physiol (2017) 8(1):1–8. doi: 10.1002/cphy.c170012 29357123 PMC6376873

[38] Levison BS Zhang R Wang Z Fu X DiDonato JA Hazen SL. Quantification of fatty acid oxidation products using online high-performance liquid chromatography tandem mass spectrometry. Free Radic Biol Med (2013) 59:2–13. doi: 10.1016/j.freeradbiomed.2013.03.001 PMC3772641 23499838

[39] Chen L Vasoya RP Toke NH Parthasarathy A Luo S Chiles E. Hnf4 regulates fatty acid oxidation and is required for renewal of intestinal stem cells in mice. Gastroenterology (2020) 158(4):985–99. doi: 10.1053/j.gastro.2019.11.031 PMC7062567 31759926

[40] Baier J Gänsbauer M Giessler C Arnold H Muske M Schleicher U. Arginase impedes the resolution of colitis by altering the microbiome and metabolome. J Clin Invest (2020) 130(11):5703–20. doi: 10.1172/JCI126923 PMC7598089 32721946

[41] Rath M Müller I Kropf P Closs EI Munder M. Metabolism *via* arginase or nitric oxide synthase: Two competing arginine pathways in macrophages. Front Immunol (2014) 5:532. doi: 10.3389/fimmu.2014.00532 25386178 PMC4209874

[42] Mao Y Shi D Li G Jiang P. Citrulline depletion by ass1 is required for proinflammatory macrophage activation and immune responses. Mol Cell (2022) 82(3):527–41. doi: 10.1016/j.molcel.2021.12.006 35016033

[43] Luk GD Yang P. Polyamines in intestinal and pancreatic adaptation. Gut (1987) 28 Suppl(Suppl):95–101. doi: 10.1136/gut.28.Suppl.95 PMC1434546 3121457

[44] Singh K Al-Greene NT Verriere TG Coburn LA Asim M Barry DP. The l-arginine transporter solute carrier family 7 member 2 mediates the immunopathogenesis of attaching and effacing bacteria. PloS Pathog (2016) 12(10):e1005984. doi: 10.1371/journal.ppat.1005984 27783672 PMC5081186

[45] Panda SK Kim D-H Desai P Rodrigues PF Sudan R Gilfillan S. Slc7a8 is a key amino acids supplier for the metabolic programs that sustain homeostasis and activation of type 2 innate lymphoid cells. Proc Natl Acad Sci USA (2022) 119(46):e2215528119. doi: 10.1073/pnas.2215528119 36343258 PMC9674248

[46] Diskin C Pålsson-McDermott EM. Metabolic modulation in macrophage effector function. Front Immunol (2018) 9:270. doi: 10.3389/fimmu.2018.00270 29520272 PMC5827535

[47] Gordon S Martinez FO. Alternative activation of macrophages: Mechanism and functions. Immunity (2010) 32(5):593–604. doi: 10.1016/j.immuni.2010.05.007 20510870

[48] Locati M Curtale G Mantovani A. Diversity, mechanisms, and significance of macrophage plasticity. Annu Rev Pathol (2020) 15:123–47. doi: 10.1146/annurev-pathmechdis-012418-012718 PMC7176483 31530089

[49] Fleetwood AJ Lawrence T Hamilton JA Cook AD. Granulocyte-macrophage colony-stimulating factor (csf) and macrophage csf-dependent macrophage phenotypes display differences in cytokine profiles and transcription factor activities: Implications for csf blockade in inflammation. J Immunol (2007) 178(8):5245–52. doi: 10.4049/jimmunol.178.8.5245 17404308

[50] Viola A Munari F Sánchez-Rodríguez R Scolaro T Castegna A. The metabolic signature of macrophage responses. Front Immunol (2019) 10:1462. doi: 10.3389/fimmu.2019.01462 31333642 PMC6618143

[51] Kelly B O'Neill LAJ. Metabolic reprogramming in macrophages and dendritic cells in innate immunity. Cell Res (2015) 25(7):771–84. doi: 10.1038/cr.2015.68 PMC4493277 26045163

[52] Van den Bossche J Baardman J Otto NA van der Velden S Neele AE van den Berg SM. Mitochondrial dysfunction prevents repolarization of inflammatory macrophages. Cell Rep (2016) 17(3):684–96. doi: 10.1016/j.celrep.2016.09.008 27732846

[53] Ji L Zhao X Zhang B Kang L Song W Zhao B. Slc6a8-mediated creatine uptake and accumulation reprogram macrophage polarization *via* regulating cytokine responses. Immunity (2019) 51(2):272–84. doi: 10.1016/j.immuni.2019.06.007 31399282

[54] Lunt SY Vander Heiden MG. Aerobic glycolysis: Meeting the metabolic requirements of cell proliferation. Annu Rev Cell Dev Biol (2011) 27:441–64. doi: 10.1146/annurev-cellbio-092910-154237 21985671

[55] Martínez-Reyes I Chandel NS. Mitochondrial tca cycle metabolites control physiology and disease. Nat Commun (2020) 11(1):102. doi: 10.1038/s41467-019-13668-3 31900386 PMC6941980

[56] Liu Y Liu Y He Y Zhang N Zhang S Li Y. Hypoxia-induced fus-circtbc1d14 stress granules promote autophagy in tnbc. Adv Sci (Weinh) (2023) 10(10):e2204988. doi: 10.1002/advs.202204988 36806670 PMC10074116

[57] Geijtenbeek TBH Gringhuis SI. Signalling through c-type lectin receptors: Shaping immune responses. Nat Rev Immunol (2009) 9(7):465–79. doi: 10.1038/nri2569 PMC7097056 19521399

[58] Wang E-J Wu M-Y Ren Z-Y Zheng Y Ye RD Tan CSH. Targeting macrophage autophagy for inflammation resolution and tissue repair in inflammatory bowel disease. Burns Trauma (2023) 11:tkad004. doi: 10.1093/burnst/tkad004 37152076 PMC10157272

[59] Kimmelman AC White E. Autophagy and tumor metabolism. Cell Metab (2017) 25(5):1037–43. doi: 10.1016/j.cmet.2017.04.004 PMC5604466 28467923

[60] Wang Y Li X Xu X Yu J Chen X Cao X. Clec7a expression in inflammatory macrophages orchestrates progression of acute kidney injury. Front Immunol (2022) 13:1008727. doi: 10.3389/fimmu.2022.1008727 36189317 PMC9520532

[61] Glick D Barth S Macleod KF. Autophagy: Cellular and molecular mechanisms. J Pathol (2010) 221(1):3–12. doi: 10.1002/path.2697 PMC2990190 20225336

[62] Siqueira M Ribeiro R Travassos LH. Autophagy and its interaction with intracellular bacterial pathogens. Front Immunol (2018) 9:935. doi: 10.3389/fimmu.2018.00935 29875765 PMC5974045

[63] Puleston DJ Buck MD Klein Geltink RI Kyle RL Caputa G O'Sullivan D. Polyamines and eif5a hypusination modulate mitochondrial respiration and macrophage activation. Cell Metab (2019) 30(2):352–63. doi: 10.1016/j.cmet.2019.05.003 PMC6688828 31130465

[64] Lim SC Tajika M Shimura M Carey KT Stroud DA Murayama K. Loss of the mitochondrial fatty acid β-oxidation protein medium-chain acyl-coenzyme a dehydrogenase disrupts oxidative phosphorylation protein complex stability and function. Sci Rep (2018) 8(1):153. doi: 10.1038/s41598-017-18530-4 29317722 PMC5760697

[65] Magalhaes-Novais S Blecha J Naraine R Mikesova J Abaffy P Pecinova A. Mitochondrial respiration supports autophagy to provide stress resistance during quiescence. Autophagy (2022) 18(10):2409–26. doi: 10.1080/15548627.2022.2038898 PMC9542673 35258392

[66] Davies LC Jenkins SJ Allen JE Taylor PR. Tissue-resident macrophages. Nat Immunol (2013) 14(10):986–95. doi: 10.1038/ni.2705 PMC4045180 24048120

