# Supplementary material for: Integrated transcriptomic and metabolomic analysis reveals the metabolic programming of GM-CSF- and M-CSF- differentiated mouse macrophages
Source: Front Immunol. 2023 Sep 25;14:1230772. doi: 10.3389/fimmu.2023.1230772 (PMC10560851; doi:10.3389/fimmu.2023.1230772)
Supplement: Supplementary file 1 [file DataSheet_1.docx]

Supplementary Material

Integrated transcriptomic and metabolomic analysis reveals the metabolic programming of GM-CSF- and M-CSF- differentiated mouse macrophages

**Qianyue Zhang^1, †^, Qiaoling Song^1,2, †^, Shan Liu^2^, Yuting Xu^1^, Danling Gao^1^, Peizhe Lu^3^, Yuantao Liu^4^, Guanghui Zhao^5, 6^, Lihong Wu^1^, Chenyang Zhao^1, ৳^, *, Jinbo Yang^1,2, *^ Correspondence:** Jinbo Yang: yangjb@ouc.edu.cn, Chenyang Zhao: zhaoc2@ccf.org

# Supplementary Figures and Tables

## Supplementary Figures

**
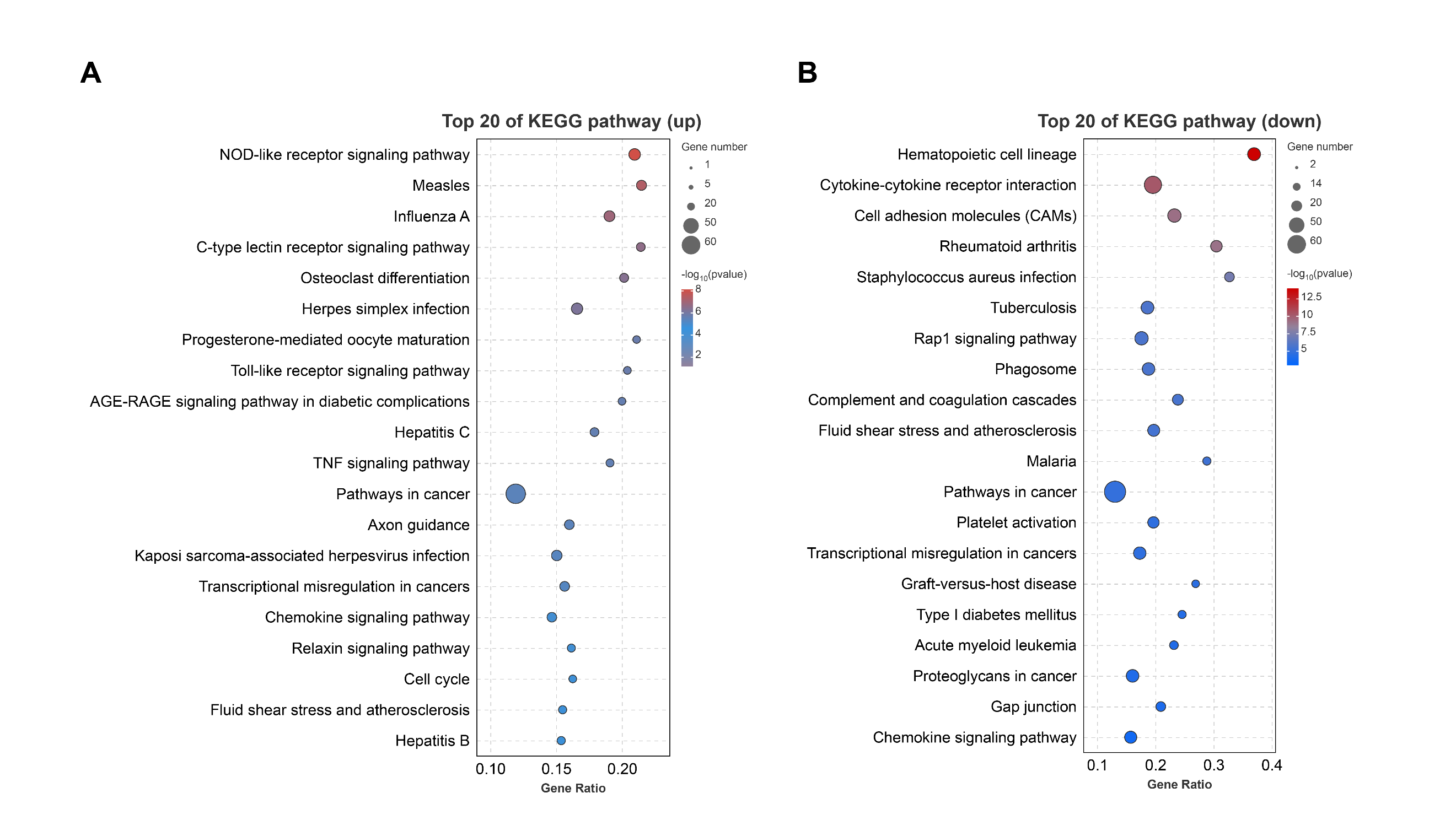
**

**Supplementary Figure 1.** **GM and M macrophages exhibit different gene expression profile.**

**(A-B)** The top 20 pathways of KEGG analysis of upregulated DEGs **(A)** and downregulated DEGs **(B)** in M compared with GM group.


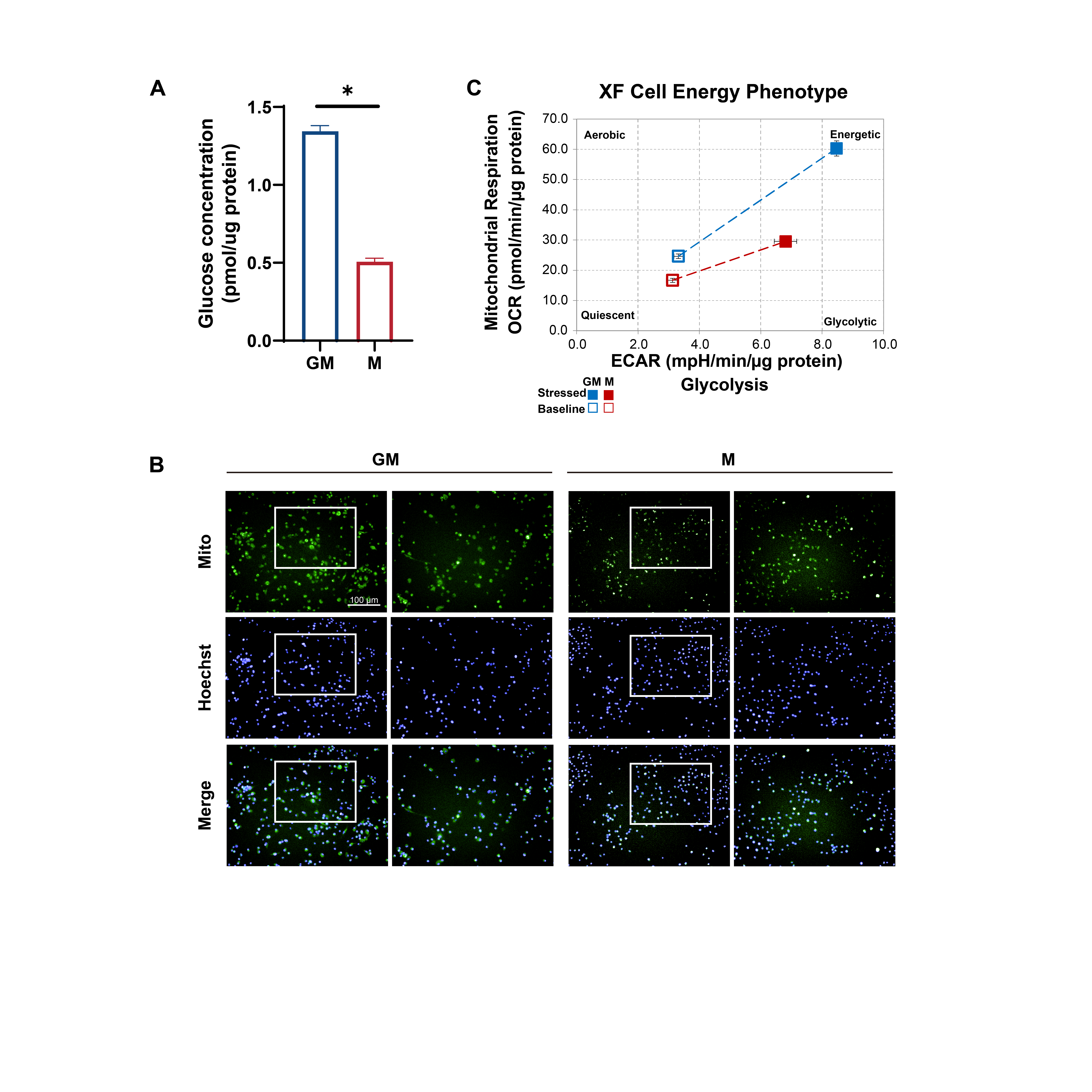


**Supplementary Figure 2. Determination of cell energy and mitochondrial contents between GM and M macrophages.**

**(A)** Glucose concentration of GM and M cells measured by the Glucose Assay Kit. n=3 for each group. *P* < 0.05 is indicated by *. **(B)** GM and M cells stained with Mito-Tracker Green to visualize the mitochondria in green and Hoechst 33342 to visualize the nucleus in cyan. Scale bars, 100 μm. The white box section was cropped and placed in Figure 2H. **(C)** OCR and ECAR of GM and M before (basal condition) and after (stressed condition) they were treated with Oligomycin and FCCP from Cell Energy Phenotype Test Kit. ECAR represents glycolysis and OCR represents mitochondrial respiration. n=5 for each group.

**
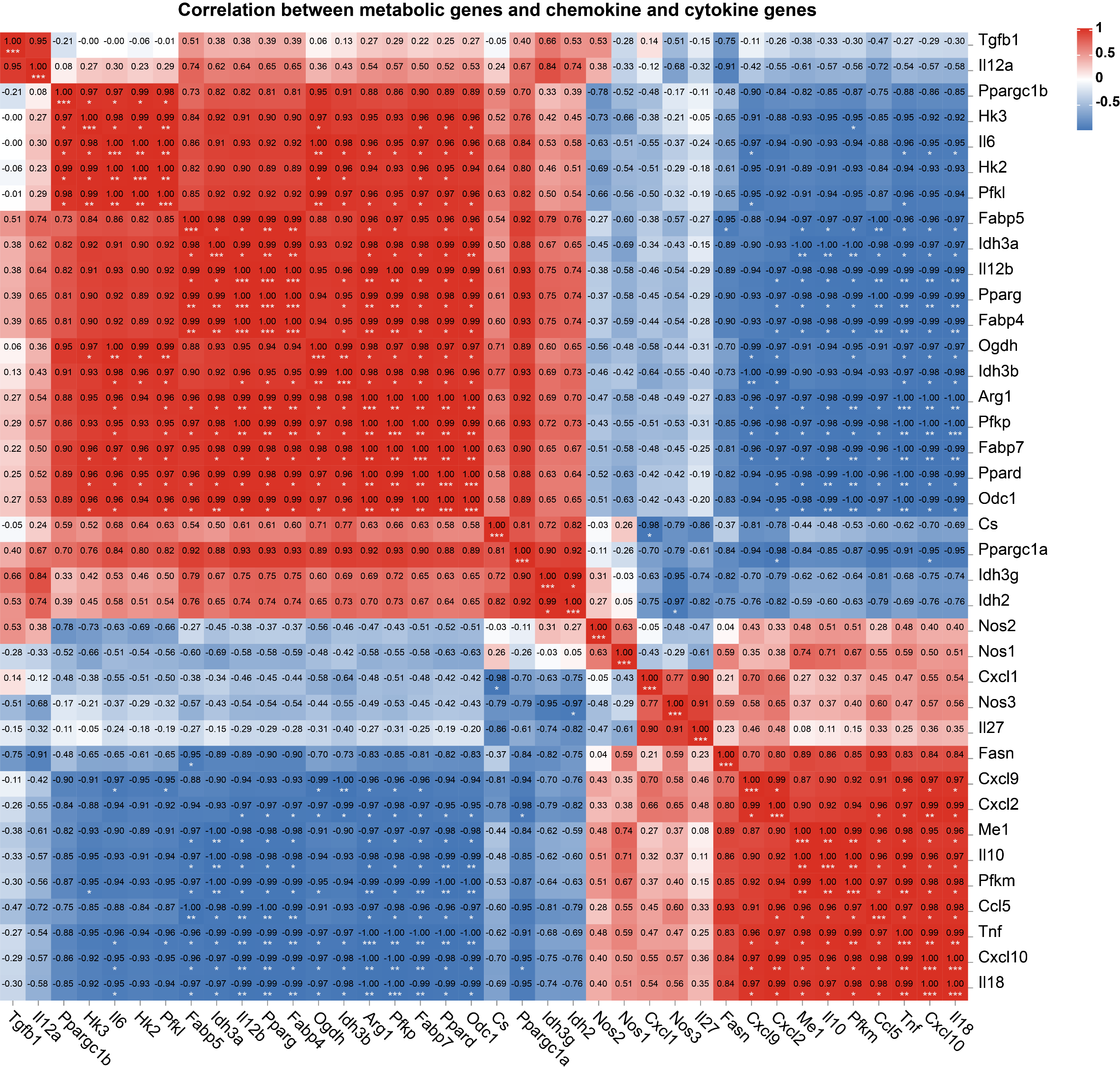
**

**Supplementary Figure 3. Heatmap of the Pearson correlation matrix between selected metabolic genes and chemokine and cytokine genes.**

Red color represents positive correlation and blue color represents negative correlation. Color intensity represents the absolute correlation values. * indicates 0.01≤ *P* < 0.05, ** indicates 0.001 < P <0.01, and *** indicates P ≤ 0.001.

**
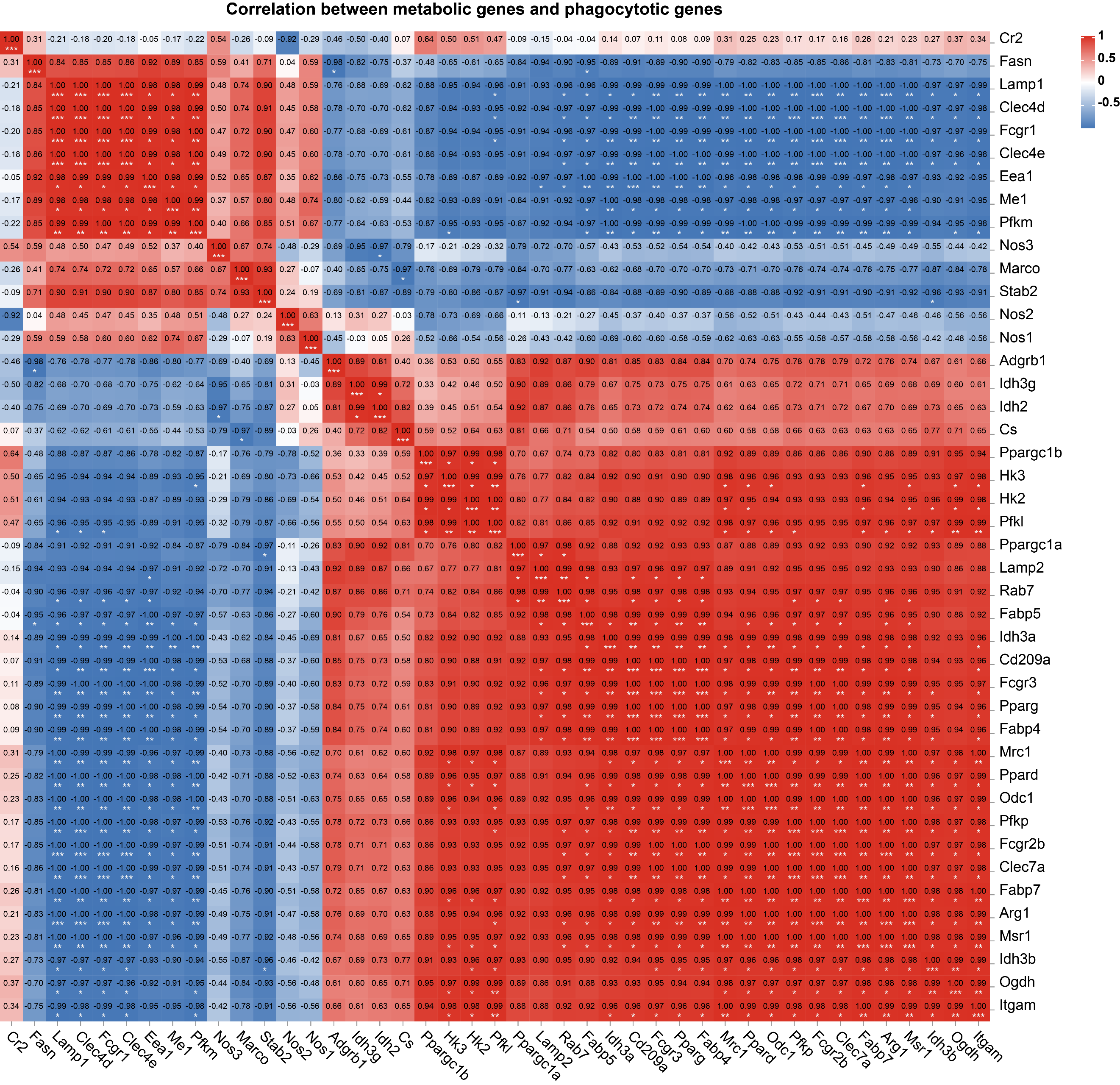
**

**Supplementary Figure 4. Heatmap of the Pearson correlation matrix between selected metabolic genes and phagocytotic genes.**

Red color represents positive correlation and blue color represents negative correlation. Color intensity represents the absolute correlation values. * indicates 0.01≤ *P* < 0.05, ** indicates 0.001 < P <0.01, and *** indicates P ≤ 0.001.

## Supplementary Tables

| **Supplementary Table 1. Top50 pairs of correlated metabolic genes and cytokine and chemokine genes** | | | |  |
| --- | --- | --- | --- | --- |
| Pathway | Meta_gene | Infla_gene | Correlation value | |
| Arginine | Arg1 | Tnf | -0.999831806 | |
| Arginine | Arg1 | Il18 | -0.996038016 | |
| Arginine | Arg1 | Cxcl10 | -0.995167852 | |
| Arginine | Arg1 | Il12b | 0.993111532 | |
| Arginine | Me1 | Tnf | 0.975387758 | |
| Arginine | Me1 | Pfkm | 0.99319865 | |
| Arginine | Me1 | Il10 | 0.997858223 | |
| Arginine | Odc1 | Tnf | -0.998391721 | |
| Arginine | Odc1 | Il10 | -0.993292509 | |
| Arginine | Odc1 | Il12b | 0.989492544 | |
| Glycolysis | Hk2 | Il6 | 0.996656834 | |
| Glycolysis | Hk3 | Il6 | 0.980928688 | |
| Glycolysis | Pfkl | Il6 | 0.998252014 | |
| Glycolysis | Pfkm | Il12b | -0.987761398 | |
| Glycolysis | Pfkm | Ccl5 | 0.968082583 | |
| Glycolysis | Pfkm | Cxcl10 | 0.976142844 | |
| Glycolysis | Pfkm | Il18 | 0.978888648 | |
| Glycolysis | Pfkm | Tnf | 0.993812388 | |
| Glycolysis | Pfkm | Il10 | 0.998454775 | |
| Glycolysis | Pfkp | Il18 | -0.999011608 | |
| Glycolysis | Pfkp | Cxcl10 | -0.998378794 | |
| Glycolysis | Pfkp | Tnf | -0.998005925 | |
| Glycolysis | Pfkp | Il12b | 0.995727893 | |
| Lipid | Fabp4 | Ccl5 | -0.994688349 | |
| Lipid | Fabp4 | Il18 | -0.991302915 | |
| Lipid | Fabp4 | Tnf | -0.990826987 | |
| Lipid | Fabp4 | Cxcl10 | -0.989229155 | |
| Lipid | Fabp4 | Il12b | 0.999739052 | |
| Lipid | Fabp5 | Il12b | 0.988013686 | |
| Lipid | Fabp7 | Tnf | -0.998758338 | |
| Lipid | Fabp7 | Il18 | -0.991570359 | |
| Lipid | Fabp7 | Cxcl10 | -0.990915071 | |
| Lipid | Fabp7 | Il6 | 0.974824537 | |
| Lipid | Fabp7 | Il12b | 0.98577512 | |
| Lipid | Ppard | Tnf | -0.997984071 | |
| Lipid | Ppard | Il10 | -0.992310811 | |
| Lipid | Ppard | Il6 | 0.963802172 | |
| Lipid | Ppard | Il12b | 0.986776725 | |
| Lipid | Pparg | Ccl5 | -0.995066862 | |
| Lipid | Pparg | Il18 | -0.992227272 | |
| Lipid | Pparg | Tnf | -0.990713131 | |
| Lipid | Pparg | Cxcl10 | -0.990294771 | |
| Lipid | Pparg | Il12b | 0.999845429 | |
| Lipid | Ppargc1b | Il6 | 0.97236472 | |
| TCA cycle | Idh3a | Il10 | -0.997520222 | |
| TCA cycle | Idh3a | Il12b | 0.98987243 | |
| TCA cycle | Idh3b | Cxcl9 | -0.9973868 | |
| TCA cycle | Idh3b | Cxcl2 | -0.988122602 | |
| TCA cycle | Idh3b | Il6 | 0.980015534 | |
| TCA cycle | Ogdh | Il6 | 0.996429386 | |

| **Supplementary Table 2. Top50 pairs of correlated metabolic genes and phagocytotic genes** | | | |
| --- | --- | --- | --- |
| Pathway | Meta_gene | Phag_gene | Correlation value |
| Arginine | Arg1 | Clec4d | -0.99944 |
| Arginine | Arg1 | Clec4e | -0.99882 |
| Arginine | Arg1 | Clec7a | 0.998886 |
| Arginine | Arg1 | Fcgr1 | -0.99924 |
| Arginine | Arg1 | Fcgr2b | 0.999185 |
| Arginine | Arg1 | Lamp1 | -0.99986 |
| Arginine | Arg1 | Msr1 | 0.999373 |
| Arginine | Odc1 | Clec4d | -0.99703 |
| Arginine | Odc1 | Clec4e | -0.9978 |
| Arginine | Odc1 | Clec7a | 0.995522 |
| Arginine | Odc1 | Fcgr1 | -0.99866 |
| Arginine | Odc1 | Fcgr2b | 0.995992 |
| Arginine | Odc1 | Lamp1 | -0.99829 |
| Arginine | Odc1 | Mrc1 | 0.996744 |
| Arginine | Odc1 | Msr1 | 0.995418 |
| Glycolysis | Pfkm | Fcgr1 | 0.995544 |
| Glycolysis | Pfkp | Clec4d | -0.99904 |
| Glycolysis | Pfkp | Clec4e | -0.99798 |
| Glycolysis | Pfkp | Clec7a | 0.999362 |
| Glycolysis | Pfkp | Fcgr1 | -0.99769 |
| Glycolysis | Pfkp | Fcgr2b | 0.999386 |
| Glycolysis | Pfkp | Lamp1 | -0.99818 |
| Glycolysis | Pfkp | Msr1 | 0.997909 |
| Lipid | Fabp4 | Cd209a | 0.999619 |
| Lipid | Fabp4 | Clec4e | -0.99549 |
| Lipid | Fabp4 | Clec7a | 0.996048 |
| Lipid | Fabp4 | Eea1 | -0.99774 |
| Lipid | Fabp4 | Fcgr2b | 0.995469 |
| Lipid | Fabp4 | Fcgr3 | 0.999602 |
| Lipid | Fabp5 | Eea1 | -0.99639 |
| Lipid | Fabp7 | Clec4d | -0.99652 |
| Lipid | Fabp7 | Clec4e | -0.99577 |
| Lipid | Fabp7 | Clec7a | 0.995075 |
| Lipid | Fabp7 | Fcgr1 | -0.99712 |
| Lipid | Fabp7 | Fcgr2b | 0.995738 |
| Lipid | Fabp7 | Itgam | 0.99576 |
| Lipid | Fabp7 | Lamp1 | -0.9986 |
| Lipid | Fabp7 | Mrc1 | 0.998145 |
| Lipid | Fabp7 | Msr1 | 0.999321 |
| Lipid | Ppard | Clec4d | -0.99595 |
| Lipid | Ppard | Clec4e | -0.99663 |
| Lipid | Ppard | Fcgr1 | -0.99782 |
| Lipid | Ppard | Lamp1 | -0.99782 |
| Lipid | Ppard | Mrc1 | 0.998059 |
| Lipid | Ppard | Msr1 | 0.995492 |
| Lipid | Pparg | Cd209a | 0.999354 |
| Lipid | Pparg | Clec4e | -0.99528 |
| Lipid | Pparg | Clec7a | 0.99617 |
| Lipid | Pparg | Eea1 | -0.99723 |
| Lipid | Pparg | Fcgr2b | 0.995573 |
| Lipid | Pparg | Fcgr3 | 0.999383 |
